# Sensory-Cell Population Integrity Required to Preserve Minimal and Normal Vestibulo-ocular Reflexes Reveals the Critical Role of Type I Hair Cells in Canal- and Otolith-Specific Functions

**DOI:** 10.1523/ENEURO.0303-25.2026

**Published:** 2026-02-19

**Authors:** Louise Schenberg, François Simon, Aïda Palou, Cassandre Djian, Michele Tagliabue, Jordi Llorens, Mathieu Beraneck

**Affiliations:** ^1^Université Paris Cité, CNRS UMR 8002, INCC - Integrative Neuroscience and Cognition Center, Paris F-75006, France; ^2^Department of Paediatric Otolaryngology, Hôpital Necker-Enfants Malades, APHP, Paris F-75015, France; ^3^Departament de Ciències Fisiològiques, Universitat de Barcelona, l’Hospitalet de Llobregat 08907, Spain; ^4^Institut de Neurociènces, Universitat de Barcelona, Barcelona 08035, Spain; ^5^Institut d'Investigació Biomèdica de Bellvitge (IDIBELL), l’Hospitalet de Llobregat 08907, Spain

**Keywords:** hair cells, thresholds, vestibular, VOR

## Abstract

Vestibular dysfunction constitutes a major medical concern, and regeneration of hair cells (HCs) is a primary target of gene therapy aimed at restoring vestibular functions. Thus far, therapeutic trials in animal models targeting vestibular loss associated with genetic diseases have yielded variable and partial results, and the functional identity and quantity of HCs required to restore minimal or normal vestibular function remain undefined. Indeed, direct comparisons between structural pathology and quantitative assessments of vestibular dysfunctions are lacking in humans and are rather limited in animal models, representing a significant gap in current knowledge. Here, we present an innovative methodology to bridge the gap between HC integrity and functional vestibular loss in individual mice of either sex. Gradual vestibular deficits were induced through a dose-dependent ototoxic lesion, quantified with canal or utricular-specific vestibulo-ocular reflex tests, and were then correlated in all individuals with the loss of type I and type II HCs in different regions of ampulla and macula. Our findings reveal that the structure–function relationship is nonlinear, with lower bound of ∼50% of HCs necessary to retain minimal vestibular function, and threshold exceeding 80% to preserve normal function, thus shedding light on population coding mechanisms for vestibular response. Our data further support the decisive role of type I, rather than type II, HC in the tested VOR functions.

## Significance Statement

Vestibular dysfunction poses a major medical challenge, with significant consequences for balance, spatial orientation, and quality of life. While regenerative therapies targeting hair cell (HC) repair offer promise, the minimal structural requirements for restoring normal vestibular functions remain unclear. Through an innovative methodology that combines precise vestibulo-ocular reflex (VOR) quantification and region-specific analyses of HC loss in mice, we demonstrate a nonlinear relationship between structural integrity and functional recovery. Our findings establish critical thresholds of HC preservation, ∼50% for minimal vestibular function and over 80% for normal function. These insights provide valuable benchmarks for translational research, refining therapeutic strategies for vestibular pathologies and advancing our understanding of population-coding mechanisms.

## Introduction

The vestibular system is fundamental to the ability of vertebrates to detect head movements and to discriminate between self-motion and environmental motion. Angular and linear head accelerations are encoded by two distinct sets of inner ear organs: the three semicircular canals, which encode rotational movements across all planes, and the otolithic organs, consisting of the saccule and utricle, which encode head accelerations relative to gravity (Angelaki and Cullen, 2008). Vestibular information is conveyed from the inner ear to the brainstem vestibular nuclei and vestibulo-cerebellum, where it is processed for various functional pathways. Thus, the vestibulo-ocular pathway is responsible for gaze stabilization, the vestibulo-spinal pathway for posture and balance, and the vestibulo-thalamic pathways for orientation and self-motion perception by providing information to subcortical and cortical circuits. Vestibular dysfunction constitutes a major medical concern, as one of its manifestations, dizziness, affects 15–35% of the general population, with a prevalence rate of up to 85% in individuals over the age of 80 (Agrawal et al., 2013). Age-related alterations of both vestibular function and the integrity of vestibular hair cells have been reported in humans (Merchant et al., 2000; Rauch et al., 2001; Lopez et al., 2005; Walther and Westhofen, 2008).

Despite its broad involvement in numerous vital functions, the initial steps of vestibular encoding remain incompletely understood and the topic of ongoing research. Vestibular sensory transduction depends on mechanoreceptors known as hair cells (HCs) located in the neuroepithelium of the different vestibular organs. These cells are characterized by their apical filiform extensions, the stereocilia, projecting from the epithelium into the lumen of the labyrinthic cavities. The HCs convert the mechanical movements induced by head motion on the stereocilia bundle into electrochemical signals for sensory input into the vestibular nerve. Vestibular HCs in mammals are categorized into distinct subtypes, each with unique morphological, molecular, electrophysiological, and synaptic characteristics (Lysakowski and Goldberg, 1997; Rüsch et al., 1998; Eatock and Songer, 2011; Eatock, 2018; McInturff et al., 2018). Type I HC found in amniotes (reptiles, birds, and mammals; Mackowetzky et al., 2021) are enveloped by a calyx-shaped terminal from a vestibular afferent neuron. These postsynaptic calyces have recently been shown to process exceptionally rapid (0.3 ms) transmission through direct electrical coupling between the hair cell and its afferent (Eatock, 2018), alongside classical quantal/glutamatergic communication (Contini et al., 2024). The type I/calyceal synapse appears particularly suited for the rapid information processing, probably essential to feed the short-delay reflexive vestibular pathways such as the vestibulo-ocular reflex (5–7 ms; Huterer and Cullen, 2002). Therefore, the encoding of rapid head motions with high-frequency content, as observed during natural head activity (Carriot et al., 2017), may primarily depend on the type I HC transducing transient, rather than sustained, stimuli (Songer and Eatock, 2013). This information would be preferentially conveyed by one of the two types of afferents defined according to electrophysiological criteria: the irregular afferents that preferentially reach the apex/center of the crista ampullaris and the central striola region of the maculae of the otolith organs (Curthoys et al., 2017).

Type II HC exhibit the greatest similarity to those observed in the cochlea and in non-mammalian vertebrates and are believed to be evolutionarily more ancient (Maudoux et al., 2022). Characterized by thinner and fewer stereocilia per hair bundle, they can be identified by their cylindrical-shaped cell body, which connects to multiple afferent bouton synapses. Type II HCs are also predominantly associated with electrophysiologically defined regular afferents, more prevalent in peripheral and nonstriolar zones of the different vestibular organs (Goldberg, 2000). Type II HC transmit sensory information exclusively through classical neurotransmitter release to their afferents and are often considered as particularly well suited to process low-frequency, tonic, and regular activity. Despite the absence of direct correspondence between type II hair cells and regular afferents, these likely constitute the two canonical presynaptic and postsynaptic elements of the peripheral pathway responsible for encoding sustained stimuli, with lower detection thresholds (Jamali et al., 2016). Notably, the presence of dimorphic terminals integrating synaptic input from both type I and multiple type II HC adds complexity to the binary connection model between HC and afferents (Eatock and Songer, 2011).

Loss of inner ear or vestibular HCs frequently occurs as a secondary ototoxic effect of aminoglycoside antibiotics (streptomycin, neomycin and gentamicin) or chemotherapy agents such as cisplatin (Halmagyi et al., 1994; Selimoğlu et al., 2003; Wu et al., 2021). In addition, studies have reported age-related changes in vestibular function (Paplou et al., 2021) as well as structural alterations in vestibular HCs in humans (Merchant et al., 2000; Rauch et al., 2001; Lopez et al., 2005; Walther and Westhofen, 2008). However, direct correlations between structural pathology and quantitative assessments of vestibular dysfunction remain rare in humans and are relatively limited in animal models (Maroto et al., 2021; Paplou et al., 2021). Despite significant development in understanding the role of the different HCs in vestibular processing, the precise relationship between HC and the resultant functional deficits remains poorly defined (Fleihan et al., 2024).

The inner ears of many non-mammalian species (e.g., amphibians, reptiles, birds) are able to produce HCs throughout their lifespan (Elliott et al., 2018). In contrast, in the mature mammalian ear, only a fraction of vestibular type II, and not type I, HCs can regenerate into an immature form from differentiating supporting cells in the extrastriolar/peripheral regions (Golub et al., 2012; Sayyid et al., 2019; Wang et al., 2019), associated with no functional recovery (Jáuregui et al., 2024). Several animal studies have attempted to investigate the restoration of vestibular function through gene therapy or pharmacological interventions aimed at promoting HC regeneration (Emptoz et al., 2017; Lahlou et al., 2024a). However, these trials have produced inconsistent and often partial outcomes, largely due to the complexity of vestibular signal processing and the heterogeneous nature of HC loss. Overall, HC regeneration remains a central goal in efforts to restore vestibular and cochlear functions (Emptoz et al., 2017).

Fundamental questions regarding the functional significance of vestibular HC persist. First, the requisite quantity or relative proportion of vestibular HC restoration necessary to achieve specified levels of functional recovery remains unknown. Second, the functional importance of the vestibular HC (e.g., type I and type II) located within various vestibular organs and epithelial regions (central/striolar zone vs periphery/extrastriolar zone) requires better characterization.

To address these gaps, we employed an innovative approach to establish a precise correlation between HC integrity and vestibular function in individual rodents (Maroto et al., 2021). In our study, vestibular deficits were gradually induced using a variety of doses of an ototoxic compound. The extent of vestibular dysfunction was quantified through canal- and utricular-specific vestibulo-ocular reflex (VOR) tests, allowing for a robust and quantitative assessment of vestibular performance. By systematically correlating these functional measurements with histological analyses of type I and type II HC loss in distinct regions of the ampullae and maculae, we aimed to elucidate the structure–function relationship governing the vestibulo-ocular reflexes.

Our findings reveal that vestibular function exhibits threshold-dependent characteristics, with a lower bound of ∼50% hair cell preservation required to sustain minimal vestibular function. In contrast, the preservation of normal vestibular function is reached at a threshold of ∼80%. These insights contribute to our understanding of population-coding strategies within the vestibular system and provide a framework for future research investigating type-specific HC regeneration. Our data further support the pivotal role of type I HC in mediating VOR functions, as their depletion was more strongly associated with vestibular deficits compared with the loss of type II HC (Schenberg et al., 2023).

## Materials and Methods

### Animals

A total of 36 male and female C57/BL6J mice, age 6–10 weeks, were used in this study. Mice were kept in standard lighting and housing conditions. Animals were used in accordance with the European Communities Council Directive 2010/63/EU. All efforts were made to minimize suffering and reduce the number of animals included in the study. All procedures were approved by the ethical committee for animal research of the Université Paris Cité.

### Head post implantation

Head post implantation surgery was performed as previously described (França de Barros et al., 2020). Mice were anesthetized with isoflurane, and a small longitudinal incision was made on their shaved head to expose the skull after a local injection of lidocaine hydrochloride (2%; 2 mg/kg). A custom-built headpost (3 × 3 × 5 mm; poly lactic acid) was first cemented (C&B Metabond; Parkell) and the sides were then covered with resin (Heraeus) for protection. Mice were placed under red light postsurgery until their full recovery and were monitored for the following 48 h.

### Ototoxic exposure

Mice were treated with a single intraperitoneal injection of IDPN. The animals were divided into five groups that received different doses of IDPN: 16 mmol/kg (*n* = 8), 24 mmol/kg (*n* = 8), 32 mmol/kg (*n* = 7), 40 (*n* = 6) mmol/kg, and the control group (*n* = 7) that received an injection of NaCl. Vestibular function was tested 2 (D14) and 3 weeks (D21) after the injection. As the longer time after exposure led to no further vestibular function deprivation (mix-model statistical test, nonsignificant differences between D14 and D21 for each test; aVOR, Hsteps, OCR, and OCR Dynamic; Extended Data Fig. 1-1), D14 and D21 functional data were pooled.

### Video-oculography recording sessions

Eye movements were recorded with a noninvasive video-oculography system (ETL 200 Iscan; acquisition rate, 120 Hz) following procedure previously described (Stahl, 2004). In brief, mice were positioned in a Plexiglass tube and head-fixed with a nose-down angle of 30° to align the horizontal semicircular canals with the yaw plane. The tube was fixed on a rotating platform on top of an extended rig with a servo-controlled motor. Eye and image/head position signals were sampled at 1 kHz, digitally recorded (CED power1401 MkII) with Spike 2 software and analyzed off-line in Matlab (Matlab, The MathWorks; RRID: SCR:001622) programming environment. Recording sessions were performed in a temperature-controlled room (21–24°) and lasted up to 45 min.

### Vestibular stimulation

Canal (VORd and Hsteps) and utricular-specific (OCR and OCR Dynamic) tests were performed in complete darkness, with the mouse surrounded by an opaque dome or box. Pilocarpine 2% was placed on the eye of the mouse before the test to prevent excessive pupil dilatation.

Sinusoidal angular rotations around the vertical axis were performed to record the horizontal angular vestibulo-ocular reflex (aVOR), at different frequencies: 0.2, 0.5, 0.8, 1, and 2 Hz at a peak velocity of 30°/s. At least 10 cycles were analyzed per frequency and the compensatory eye movements were quantified by calculating the gain (ratio between the eye velocity and table velocity) and the phase (normalized latency between the eye and the table velocities; Carcaud et al., 2017). For IDPN-treated mice, low (typically <0.10) values of gain associated with VAF (variance-accounted-for) <0.5 have abnormal phase values. Following Schenberg et al.’s (2023) methodology, those artifactual values were specifically reported in gray (Extended Data Fig. 1-1*E*) and were discarded from the statistical analysis of the aVOR phase.

Hsteps tests were performed using an abrupt acceleration followed by a sudden stop after three 360° rotations with a constant-velocity of 50°/s on the yaw plane (acceleration 250°/s^2^). The Hsteps gain was calculated as the ratio between the peak velocity of the slow phases at onset and offset of rotations and the rotation velocity.

**Figure 1.**
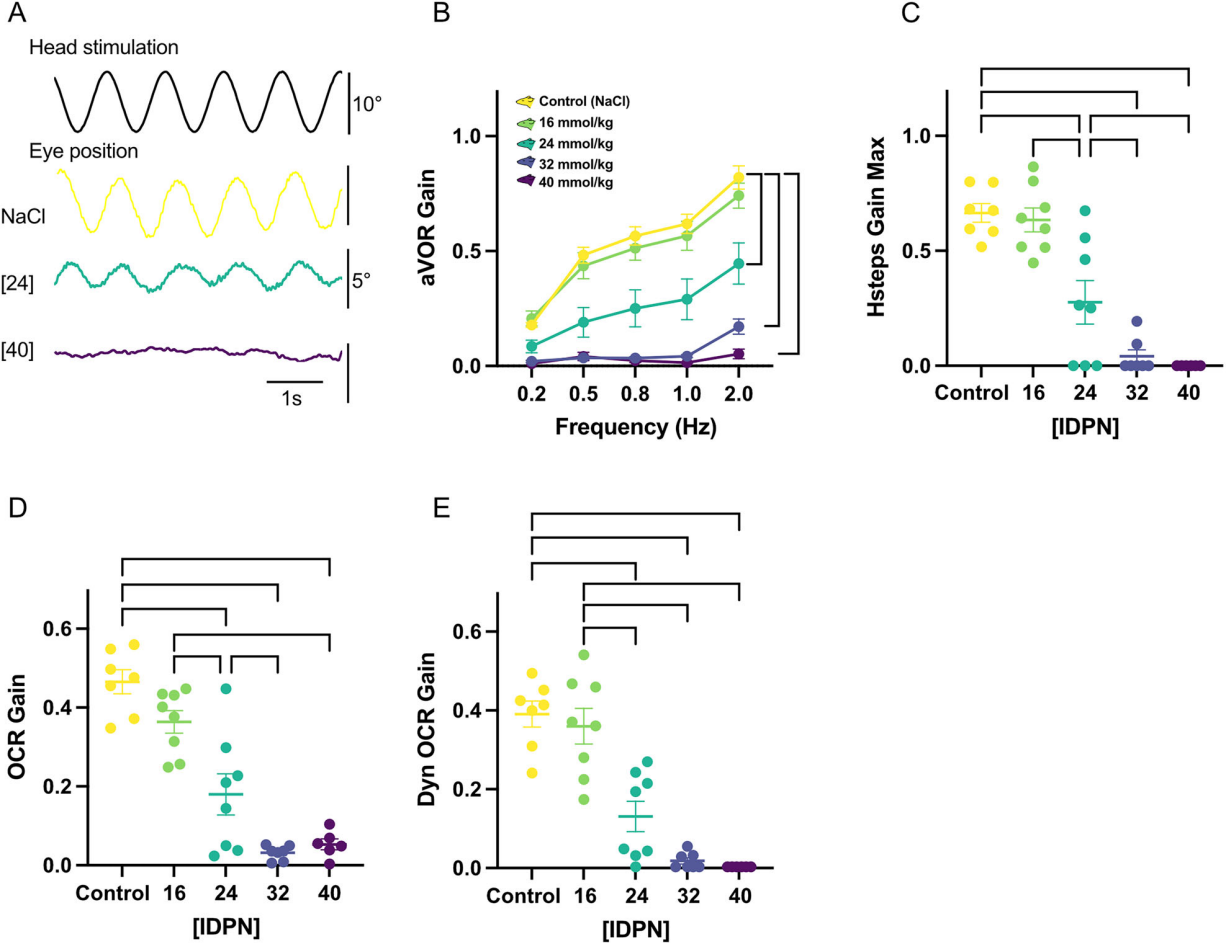
***A***, Typical raw traces of compensatory eye movements evoked by a 1 Hz, 30°/s angular sinusoidal rotations in three mice from the control, 24 and 40 mM groups. ***B***, Bode plot of the gain of the horizontal angular vestibulo-ocular reflex at different frequencies (tested with sinusoidal rotations at peak velocity 30°/s) for the five different concentration tested. ***C***, Maximal gain response in respond to abrupt horizontal acceleration/deceleration (steps of acceleration from 0 to 50°/s, with peak velocity 250°/s^2^). ***D***, Gain of the ocular counter roll reflex measured during static roll in range ±0–40. ***E***, Dynamic ocular counter roll reflex gain. The gain was calculated using the vertical component of the compensatory eye movement evoked in response to off-vertical axis rotation with a tilt angle of 17°. In all panels, sample sizes are as follows: [Control] (*n* = 7), [16] (*n* = 8), [24] (*n* = 8), [32] (*n* = 7) and [40] (*n* = 6). Vertical and horizontal brackets indicate significant differences (*p* < 0.05). Values represented mean ± SEM (see also Extended Data Fig. 1-1).

Static Counter Roll (OCR) tests were performed first by measuring the vertical pupil position according to the head tilt angle at 0° in the horizontal plane. The table was then tilted from left to right in incremental steps of 10° (from 0 to 40°), with static periods of at least 10 s between oscillations (Fig. 1*D*) to record stable eye position. The OCR gain corresponds to the slope of the linear regression of the vertical eye angle and the head tilt angles (Oommen and Stahl, 2008; Simon et al., 2020).

10.1523/ENEURO.0303-25.2026.f1-1 Figure 1-1 Mean aVOR gain (A), HStep gain (B), static OCR gain (C), Dynamic OCR gain (D) of all (n = 36) mice at D14 and D21 after injection. E) aVOR Phase values at different frequencies for the 5 different concentration tested. Abnormal phase values associated with gain<0.1 and VAF<0.5 are represented in grey. Sample size are [Control] (n = 7), [16] (n = 8), [24] (n = 8), [32] (n = 7) and [40] (n = 6). Download Figure 1-1, TIF file.

Dynamic OCR tests were performed with the vestibular turntable tilted with a 17° off-axis angle (Beraneck et al., 2012; Idoux et al., 2018; Simon et al., 2021). Then, 50°/s continuous stimulations were performed in a counterclockwise and then clockwise direction. The Dynamic OCR gain corresponds to the dynamic or vertical component of the compensatory eye movement divided by the maximal differential tilt angle (i.e., 34°).

### Immunolabeling of the hair cells in the horizontal semicircular canals and the utricle

The inner ear of all mice tested with vestibular function tests (*n* = 36) were then used to perform immunofluorescence analysis on hair cells in the vestibular endorgans. Mice were anesthetized with an overdose of intraperitoneal injection of ketamine hydrochloride (10%)/xylazine (5%) and decapitated. The histology was done following previously published protocol of Maroto et al. (2021). The vestibular epithelia were dissected and fixed for 1 h in a 4% solution of paraformaldehyde (PFA). PFA was washed twice with phosphate-buffered saline (PBS) and the samples were placed in a cryoprotective solution at 4° for 2 h for effective embedding and then stored at −20°. Before the immunochemistry, samples (one horizontal canal and one utricle) were put at room temperature and rinsed twice in PBS. While under slow agitation, the samples were incubated twice, first for 1 h with 4% Triton X-100 (Sigma-Aldrich) in PBS to permeabilize the tissue and a second time for 1 h in 0.5% Triton X-100 1% fish gelatin (CAS #9000-70-8, Sigma-Aldrich) in PBS to block nonspecific binding sites. The incubation with the primary antibodies was then performed in 0.1% Triton X-100, 1% fish gelatin in PBS at 4° for 24 h. After rinsing, the secondary antibodies were incubated in the same conditions. The secondary antibodies were rinsed and the vestibular epithelia were mounted on slides with Fluoromount (F4680, Sigma-Aldrich). The slides were visualized with a Zeiss LSM880 confocal microscope and one square image (67.48 × 67.48 µm) was obtained from each area to be counted, using an objective of 63× (NA:1,4) at zoom 2. To properly analyze the whole thickness of the vestibular epithelium, *Z*-stacks of 0.5 μm were obtained and observed with ImageJ (National Institute of Mental Health). The primary antibodies used were mouse anti-Myosin VIIa (Myo7a; Developmental Studies Hybridoma Bank, clone 138-1-s, RRID: AB_2282417, 1:100), guinea pig anti-calretinin (Synaptic Systems, #214-104, RRID: AB_10635160, 1:500), rabbit anti-oncomodulin (Swant, OMG4, RRID:AB_10000346, 1:400) and goat anti-osteopontin (SPP1; R&D Systems, #AF08, RRID: AB_2194992, 1:200). Their respective secondary antibodies were DyLight 405 donkey anti-rabbit ifG H + L (Jackson ImmunoResearch, #711-475-152), Alexa Fluor 488 donkey anti-guinea pig IgG H + L (Jackson ImmunoResearch, #706-605-148), Alexa Fluor 555 donkey anti-goat IgG H + L (Invitrogen, #A21432), and Alexa Fluor 647 donkey anti-mouse IgG H + L (Invitrogen, # A31571).

The oncomodulin label was used to identify the central/striola (oncomodulin+) and peripheral (oncomodulin−) regions of the organs (Hoffman et al., 2018; Maroto et al., 2021). In the utricle, images of the lateral and medial periphery (Ji et al., 2022) were obtained in the external and internal sides, respectively, of the half-moon shaped striola region. For each functional area, the images were obtained from approximately the same location in all animals. The number of total hair cells per image was assessed with the cytoplasmic labeling of the anti-Myo7a antibody (Hasson et al., 1997). Type I hair cells were labeled using anti-SPP1, mostly found in the neck zone of the cell (McInturff et al., 2018). Type II hair cells were distinguished by the colocalization of Myo7a and calretinin (Dechesne et al., 1991). A thorough evaluation of the specificity of these markers concluded that SPP1 identifies 100% of type I and calretinin identifies 85–90% of type II hair cells with no significant overlap in the adult rat (Borrajo et al., 2024). Cell type identities were ascertained by examination of these markers throughout the entire *Z*-stack.

### Statistical analysis

To test the main effect of the between-individual factor “Dose” (0, 16, 24, 32 or 40 mmol/kg) on Hsteps, OCR and DYN OCR, as well as the effect of IDPN exposure on the hair cells, a one-way analyses of variance (ANOVA) was used. For the aVOR, a two-way ANOVA was used to be able to evaluate also the main effect of the within-individual independent factor “Frequency” (0.2, 0.5, 0.8, 1, and 2 Hz) and its interaction with the Dose. The difference between D14 and D21 vestibular reflex tests was reported with a mixed model analysis. The significance threshold was set at *p* < 0.05 and Tukey’s multiple-comparison test was performed if the main effect, or an interaction, was reported significant. Prior to selecting the appropriate statistical procedures, the distribution of each dataset was evaluated using the Shapiro–Wilk test for normality. Among the 20 datasets examined, four did not satisfy the assumption of normality (*p* < 0.05). For these non-normally distributed datasets, we employed the nonparametric Kruskal–Wallis test. For datasets exhibiting normal distributions, we conducted one-way (ANOVA) to assess group effects. When significant main effects were identified, we performed post hoc multiple-comparison tests with appropriate adjustments (respectively, Dunn's or Tukey's), and we calculated effect size Cohen's and small sample-corrected Hedges’ *g* (−0.2 > *d* or *g* > 0.5 small; less than −1 large effect).

To describe the relationship between cell staining and the behavioral measures, data were first normalized expressing each value as percent of the mean value of the control group. Then, 2D nonlinear (sigmoidal) models were used to fit the datasets. Given the presence of noise on both types of measurement (function and cell counting data), the fitting required orthogonal distance regression and the fit was optimized for each dataset.

We performed hierarchical agglomerative clustering in MATLAB (Statistics and Machine Learning Toolbox, R2023b). Variables were processed by column. Pairwise Euclidean distances were computed and clustered using complete linkage. We cut the tree to define *k* = 3 clusters (cluster with MaxClust = 3). Cluster quality was assessed by the mean silhouette (0.516), and the dendrogram's cophenetic correlation was reported (0.854).

### Code and data accessibility

The code and data are freely available as Extended Data and at Mendeley Data, https://doi.org/10.17632/sbd4dnp8wh.1.

10.1523/ENEURO.0303-25.2026.d1 Data 1 Download Data 1, RTF file.

## Results

### Dose-dependent effects of the treatment on the canal- and otolith-dependent VOR

To investigate the dose–response effect of the ototoxic substance 3,3′-iminodiproprionitrile (IDPN), we performed in adult C57Bl6/J mice a single intraperitoneal injection of IDPN at increasing doses: 0 (Control, *n* = 7), 16 (*n* = 8), 24 (*n* = 8), 32 (*n* = 7) and 40 (*n* = 6) mmol/kg. Organ-specific vestibular function was quantified using gaze stabilizing reflexes tests before the injection, then 2 and 3 weeks after. There was no significant difference between vestibular function observed at D14 and D21 (mixed model analysis; Extended Data Fig. 1-1*A–D*), confirming that the IDPN-induced toxicity is stable after 2 weeks (Llorens et al., 1993; Soler-Martin et al., 2006). To reduce measurement noise, data from D14 and D21 are pooled in the rest of the manuscript.

Horizontal canal function was assessed with two complementary tests. First, horizontal sinusoidal rotations at different frequencies were performed in the dark to record the angular horizontal vestibulo-ocular reflex (aVOR; Fig. 1*A*, raw traces). The gain of aVOR is shown in the Bode plots of Figure 1*B* for the different groups and tested frequencies, and corresponding phase is reported in Extended Data Figure 1-1*E*. IDPN exposure at different doses (abbreviated as [X] mmol/kg) leads to a significant decrease of the aVOR gain for concentrations above [16], with a dose-dependent effect size (interaction dose × frequencies *F*_(16,124)_ = 15.4, *p* < 0.0001; see statistics in Table 1; Cohen's *d* and Hedges’ *g* (small sample-corrected Cohen's *d*) effect size; see statistics in Table 2). The aVOR gain of the [24] group is decreased by ∼50% compared with the Control group ([Control] vs [24]; at 0.2 Hz, *p* = 0.0625; 0.5 Hz, *p* = 0.0147; 0.8 Hz, *p* = 0.0351; 1 Hz *p* = 0.0454 and 2 Hz *p* = 0.0261) and is significantly reduced by >90% at all frequencies for the two highest doses of [32] and [40] (*p* < 0.001). There are no statistically significant differences in aVOR phase values across concentrations [0] to [24] (interaction dose × frequencies *F*_(8,65)_ = 2.242, *p* = 0.035, post hoc tests all ns). Phase values associated with higher concentrations were excluded from analysis due to their low variance-accounted-for (VAF), resulting in abnormal values unsuited for statistical comparisons. The second test, Hsteps, consisting in a single abrupt horizontal acceleration (peak acceleration 500°/s^2^; Fig. 1*C*) shows a significant decrease of gain similar to aVOR for the three highest doses (*F*_(4,31)_ = 28.58, *p* < 0.0001; [Control] vs [16] *p* = 0.9956; [Control] vs [24], [Control] vs [32] and [Control] vs [40], *p* < 0.001). While the [32] and [40] groups have no residual response, the [24] group show an intermediate VOR loss ([16] vs [24] *p* < 0.001; [24] vs [32] *p* = 0.0485).

**Table 1.** Statistical tables for the five frequencies tested of the aVOR (Fig. 1)

NaCl	16	24	32	40	0.2 Hz
	0.915	0.062	***	***	NaCl
		0.078	**	**	16
			0.227	0.14	24
				0.323	32
					40
NaCl	16	24	32	40	0.5 Hz
	0.951	*	***	***	NaCl
		0.077	**	***	16
			0.219	0.258	24
				0.997	32
					40
NaCl	16	24	32	40	0.8 Hz
	0.922	*	***	***	NaCl
		0.107	***	***	16
			0.154	0.13	24
				0.795	32
					40
NaCl	16	24	32	40	1 Hz
	0.954	*	***	***	NaCl
		0.141	***	***	16
			0.134	0.088	24
				*	32
					40
NaCl	16	24	32	40	2 Hz
	0.823	*	***	***	NaCl
		0.094	***	***	16
			0.103	*	24
				0.072	32
					40

Adjusted *p* values (Tukey-corrected) for multiple comparisons of a mixed model analysis of aVOR values (interaction dose × frequencies *F*_(16,124)_ = 15.4, *p* < 0.0001). Asterisks denote statistical significance differences (*p < 0.05, **p < 0.001, ***p < 0.0001).

**Table 2.** Cohen's *d* and small sample-corrected Hedges’ *g* (Fig. 1)

Top									
16	24	32	40	0.2Hz VOR	16	24	32	40	0.5Hz VOR
0.4	−1.57	−7.44	−8.26	NaCl	−0.35	−1.99	−6.83	−6.03	NaCl
16	24	32	40	0.8Hz VOR	16	24	32	40	1Hz VOR
−0.41	−1.73	−7.10	−6.92	NaCl	−0.35	−1.66	−7.28	−7.39	NaCl
16	24	32	40	2Hz VOR					
−0.54	−1.81	−5.75	−7.33	NaCl					
16	24	32	40	HSteps	16	24	32	40	OCR
−0.23	−1.85	−6.69	−8.36	NaCl	−1.25	−2.35	−7.36	−6.43	NaCl
16	24	32	40	Dyn Tilt					
−0.28	−2.61	−5.9	−6.05	NaCl					
Bottom									
16	24	32	40	0.2Hz VOR	16	24	32	40	0.5Hz VOR
0.38	−1.48	−6.97	−7.68	NaCl	−0.33	−1.87	−6.39	−5.61	NaCl
16	24	32	40	0.8Hz VOR	16	24	32	40	1Hz VOR
−0.39	−1.63	−6.65	−6.44	NaCl	−0.33	−1.56	−6.82	−6.87	NaCl
16	24	32	40	2Hz VOR					
−0.51	−1.7	−5.38	−6.82	NaCl					
16	24	32	40	HSteps	16	24	32	40	OCR
−0.22	−1.75	−6.26	−7.77	NaCl	−1.18	−2.21	−6.89	−5.99	NaCl
16	24	32	40	Dyn Tilt					
−0.26	−2.46	−5.51	−5.62	NaCl					

(Top) Cohen's *d* and (Bottom) Hedges’ *g* (small sample-corrected Cohen's *d*) values (−0.2 > *d* or *g* > 0.5 small; <−1 large effect).

Specific otolithic (utricular) function was quantified using both static ocular counter roll (OCR) measured during static head tilts and dynamic OCR quantified during off-vertical axis rotation (i.e., vertical component of eye movement during off-vertical axis rotation; Beraneck et al., 2012). Similarly to its effect on canal function, IDPN leads to a significant decrease of both the static OCR (Fig. 1*D*) and dynamic OCR (Fig. 1*E*) gain for the three highest doses (static OCR *F*_(4,31)_ = 32.15, *p* < 0.0001, [Control] vs [24], [Control] vs [32] and [Control] vs [40], *p* < 0.001; Dyn OCR *F*_(4,31)_ = 30.3, *p* < 0.0001, [Control] vs [24], [Control] vs [32], and [Control] vs [40], *p* < 0.001) and an intermediate loss for the [24] group (static OCR [16] vs [24], *p* = 0.0021; [24] vs [32], *p* = 0.023; Dyn OCR [16] vs [24], *p* < 0.001; [24] vs [32], *p* = 0.128).

Taken together, these results reveal the dose-dependent effect of IDPN on the canal- and otolith-specific VORs in mice.

### Dose-dependent effects of the treatment on the vestibular hair cells

**Figure 2.**
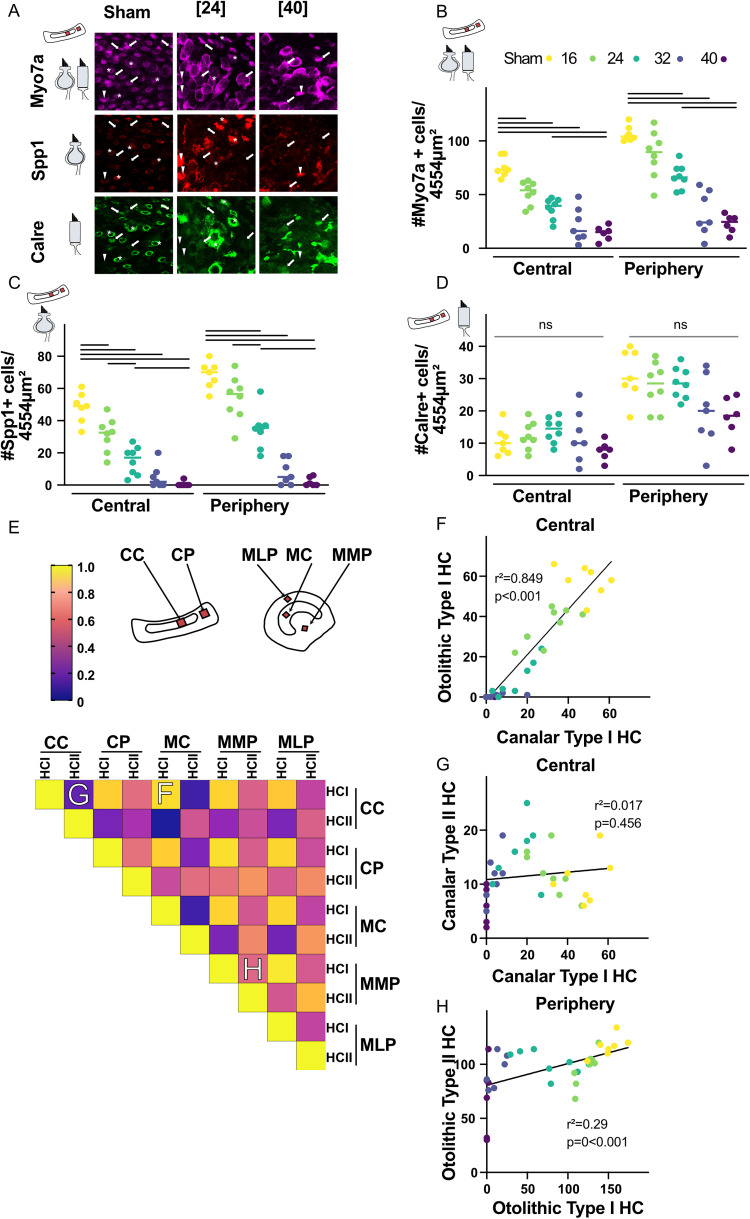
***A***, Immunolabeling of the vestibular sensory epithelium with antibodies against Myo7a, Spp1, and calretinin + in the central ampulla of the horizontal canal in SHAM [0] (left panels) and mice injected with [24] (middle panels) or [40] mmol/kg of IDPN. Samples were obtained at D21 following IDPN injection. Type II HCs (arrows) are Myo7a+ and Calre+ but lack Spp1 label. Type I HCs are Myo7a+ and Spp1+ and are encased by calyx afferents that may (asterisks) or may not (arrowheads) express calretinin. The images shown here are one single plane of the confocal stack that was used for cell counts. ***B–D***, Cell count of hair cells per image in the central and peripheral horizontal ampulla for all IDPN doses, with (***B***) Myo7a+ marker (all HC), (***C***) Spp1+ marker (type I-specific marker), (***D***) Calre+ marker (type II-specific). Sample size Sham (*n* = 7), [16] (*n* = 8), [24] (*n* = 8), [32] (*n* = 7), and [40] (*n* = 6). Horizontal brackets indicate significant differences (*p* < 0.05). Values represent mean ± SEM. ***E***, Top panels, The color code in left represents the values of the coefficient. Middle and right panels, The red schemes show the approximate locations of the central and peripheral regions where the confocal images were obtained for HC counting (CC, crista central; CP, crista periphery; MC, macula central; MMP, macula medial periphery; MLP, macula lateral periphery). Bottom panel, Correlation matrix of the *r* Pearson’s coefficient of the number of HCI and HCII for the *n* = 36 mice in different regions of ampulla and macula. ***F***, Relation between number of type I HC in the macula and the number of type I HC in the ampulla in the central region of vestibular organs of individuals. The linear fit is represented. ***G***, ***H***, Relation between number of type II HC in the ampulla and the number of type I HC in the ampulla in the central (***G***) or peripheral (***H***) regions of individuals. The linear fit is represented (see also Extended Data Fig. 2-1).

To quantify the effect of the IDPN treatment on the vestibular HC, the vestibular organs were removed and fixed in paraformaldehyde (4%) after VOR measurements at D21. Immunochemistry was used to identify type I and type II HC with type-specific markers [respectively, osteopontin (SPP1) and calretinin (Calre); Borrajo et al., 2024]. The total number of HC, independently of their identity, was quantified using the pan-hair cell marker Myo7a. Figure 2*A* shows typical examples of confocal (Zeiss LSM 880) immunostaining data obtained from [Control], [24], and [40] groups.

10.1523/ENEURO.0303-25.2026.f2-1 Figure 2-1 (A)Cell count of calyx-only type I HC in the crista and utricle. (B,C,D) Cell count of hair cells in the central and peripheral utricle for all IDPN doses, with (A) Myo7a + marker (all HC) (B) Spp1 + marker (type I specific marker), (C) Calre + marker (type II specific). Sample size Sham (n = 7), [16] (n = 8), [24] (n = 8), [32] (n = 7) and [40] (n = 6). Download Figure 2-1, TIF file.

The number of HC counts per image are plotted in Figure 2*B–D*, respectively, for Myo7a (all HC), SPP1 (type I-specific), and Calre (type II-specific) for both central and peripheral part of the horizontal crista (see Extended Data Fig. 2-1 for otolith cell counting). IDPN exposure leads to a significant decrease in the number of Myo7a-labeled HC in the central part for all doses and in the peripheral region for doses above [16] (Fig. 2*B*). The [24] treatment reduces by ∼50% Myo7a+-labeled cells compared with the Control group (one-way ANOVA, central: [Control] vs [24], *p* < 0.0001; peripheral: [Control] vs [24], *p* < 0.0001). The two highest doses, though not different from each other (central: [32] vs [40] and peripheral: [32] vs [40] *p* > 0.99), lead to a further 25% decrease in the number of Myo7a+ cells compared with the [24] group (central: [24] vs [40] *p* = 0.002; peripheral: [24] vs [40] *p* < 0.0001).

The decrease in the number of vestibular type I HC labeled by SPP1 is shown in Figure 2*C* with a similar trend than the Myo7a labeling: loss is significant for all doses >[16] in the central and the peripheral regions (central: [Control] vs [24] *p* < 0.0001 and peripheral: [control] vs [24], *p* = 0.0002). We also quantified the effect of IDPN on central calyx-only type I HC (Extended Data Fig. 2-1*A*). We noticed a similar graded loss of calyx-only type I HC in the crista and utricle (Kruskal–Wallis, crista [Control] vs [24] *p* = 0.0032; vs [30] and [40] *p* = 0 < 0.001; one-way ANOVA, utricle [Control] vs [24] to [40] *p* < 0.0001). In contrast, IDPN did not lead to any change in the number of type II HC (Calre-labeled cells) in the central part of the crista and to a small and nonsignificant decrease limited to the highest dose of 40 mmol/kg in the peripheral region (central: [Control] vs [40] *p* > 0.99; peripheral: [Control] vs [40] *p* = 0.0596).

To determine how the HC loss compares in both organs for all individuals, we compared type I and type II HC in all regions (Fig. 2*E*, top panel) by plotting a heat map based on the Pearson’s correlation matrix (Fig. 2*E*, bottom panel). While the correlation between type I HC remaining after IDPN is high in all regions, the correlation of type II HC with type I HC is low. Figure 2, *F–H*, illustrates the different sorts of correlation found between the HC identity and regions. The high correlation between the remaining type I HC in the otolith and canal organs (central: *r*^2^ = 0.849, *p* < 0.001; Fig. 2*F*) shows that the IDPN treatment leads to a highly correlated loss in both structures for all doses. However, there is no correlation between the loss of type I and type II HC in the central regions of either organ (canal: *r*^2^ = 0.017, *p* = 0.456; Fig. 2*G*) and a small yet significant correlation in the peripheral regions (otolithic *r*^2^ = 0.29, *p* < 0.001; Fig. 2*H*) mostly driven by the small decrease in type II HC at the highest dose. This analysis also shows that the ototoxicity similarly affects canals and otoliths. Overall, the effect of the IDPN treatment leads to a largely specific dose-dependent loss of type I HC, with a comparable effect in central and peripheral regions of the ampulla and utricle.

### Organ-specific structure–function relationship

**Figure 3.**
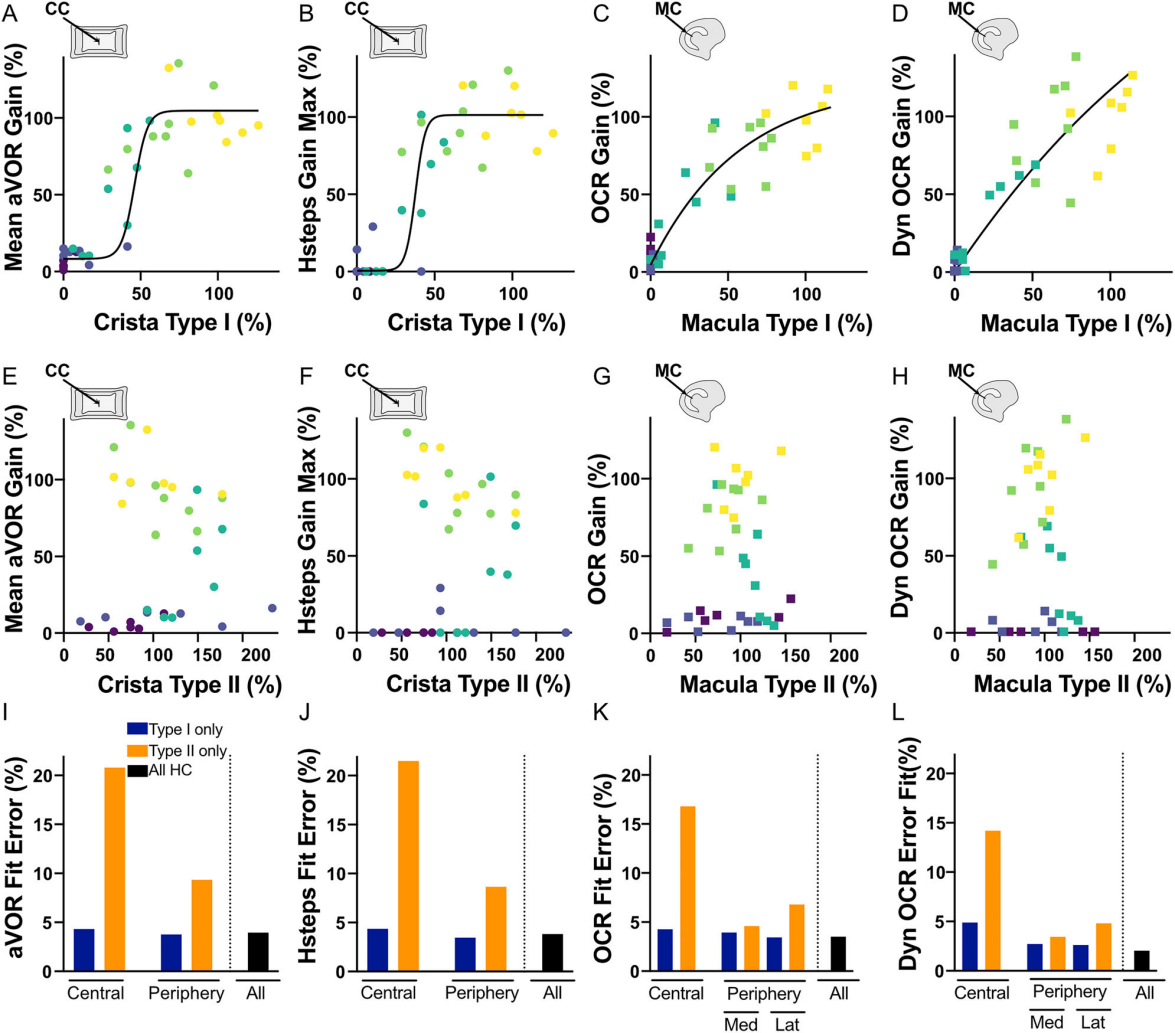
***A–D***, Normalized Mean aVOR (***A***), Hsteps (***B***), OCR (***C***), OCR Dyn (***D***), as a function of the normalized number of type I hair cells in the central part of the ampulla (***A***, ***B***) or the central part of the macula (***C***, ***D***). The nonlinear 2D fit is represented. Normalized values correspond to the gains and HC counts expressed as percentage of the respective mean value in the control group. ***E–H***, Normalized Mean aVOR (***E***), Hsteps (***F***), OCR, (***G***) OCR Dyn (***H***), as a function of the normalized number of type II hair cells in the central part of the ampulla (***E***, ***F***) or the central part of the macula (***G***, ***H***). The nonlinear 2D fit is represented. ***I–L***, Error Fit (%) of the 2D sigmoid fit for the Mean aVOR (***I***), Hsteps (***J***), OCR (***K***), OCR Dyn (***L***) in the central region and peripheral regions of the ampulla (***I***, ***J***) or the macula (***K***, ***L***) for type I HC, type II HC, and all types (type I + type II).

To investigate the relation between type I HC population of the different organs and the related vestibular function, we first compared the normalized individual gains of the vestibulo-ocular reflexes and the normalized number of type I (SPP1-labeled cells) HC in the central region of the canals (Fig. 3*A*,*B*) and otoliths (Fig. 3*C*,*D*).

Normalization values were obtained by transforming the raw data shown in Figures 1 and 2 as percentage of the corresponding mean value in the control group. The relationship between the number of type I HC and the vestibular functions was approximated by interpolating the data with 2D sigmoidal curves accounting for both the vertical (measured function values) and horizontal (HC staining data) variability. Figure 3, *A–D* (Fig. 3*A*,*B*, canals; Fig. 3*C*,*D*, otoliths), shows that the nonlinearity of the HC–function relationship is particularly evident for canal tests. Figure 3, *E–H*, illustrates the absence of correlation between the gains measured and the number of type II HC counted in the central regions of both vestibular organs (Fig. 3*E*,*F*, canals; Fig. 3*G*,*H*, otoliths).

Since IDPN treatment differentially affects specific regions and cell types (Fig. 2), we calculated the different Fit Error (%) of the sigmoids to determine which region/cell type better relates to the various VOR tests. This parameter is plotted for each test, and for the central and peripheral regions of each organ, considering either type I (SPP1 marker), type II (Calre marker), or both HC (type I + type II; Fig. 3*I*, aVOR; Fig. 3*J*, HSteps; Fig. 3*K*, OCR; Fig. 3*L*, Dyn OCR). In all instances, models based on type I HC always better approximate the function than models based on type II HC (i.e., make less error; compare blue and orange plots). Models based on type II HC in the periphery perform better than models based on type II HC in central parts, which probably relates to the partial and small dose-dependent effect found for the highest concentrations of IDPN (Fig. 2*D*,*G*,*H*). Finally, using a model summing central and peripheral type I and type II HC led to a similar (Fig. 3*I–K*) or smaller (Fig. 3*L*) error and thus better represented all the different VOR tests (Fig. 3*I–L*, compare dark and colored plots in panels).

Overall, these results demonstrate that the responses to these different VOR tests strongly depend on the integrity and proportion of type I HC. In the instances where all type I HC are lost, the presence of type II HC alone does not allow to preserve the function. These targeted analyses also suggested that the different VOR tests have differential dependency on the amount of HC preserved. We therefore decided to investigate which quantity of total HC is necessary for the maintenance of minimal or normal VOR function.

### Thresholds of minimal and normal population coding in canal and otolith functions

**Figure 4.**
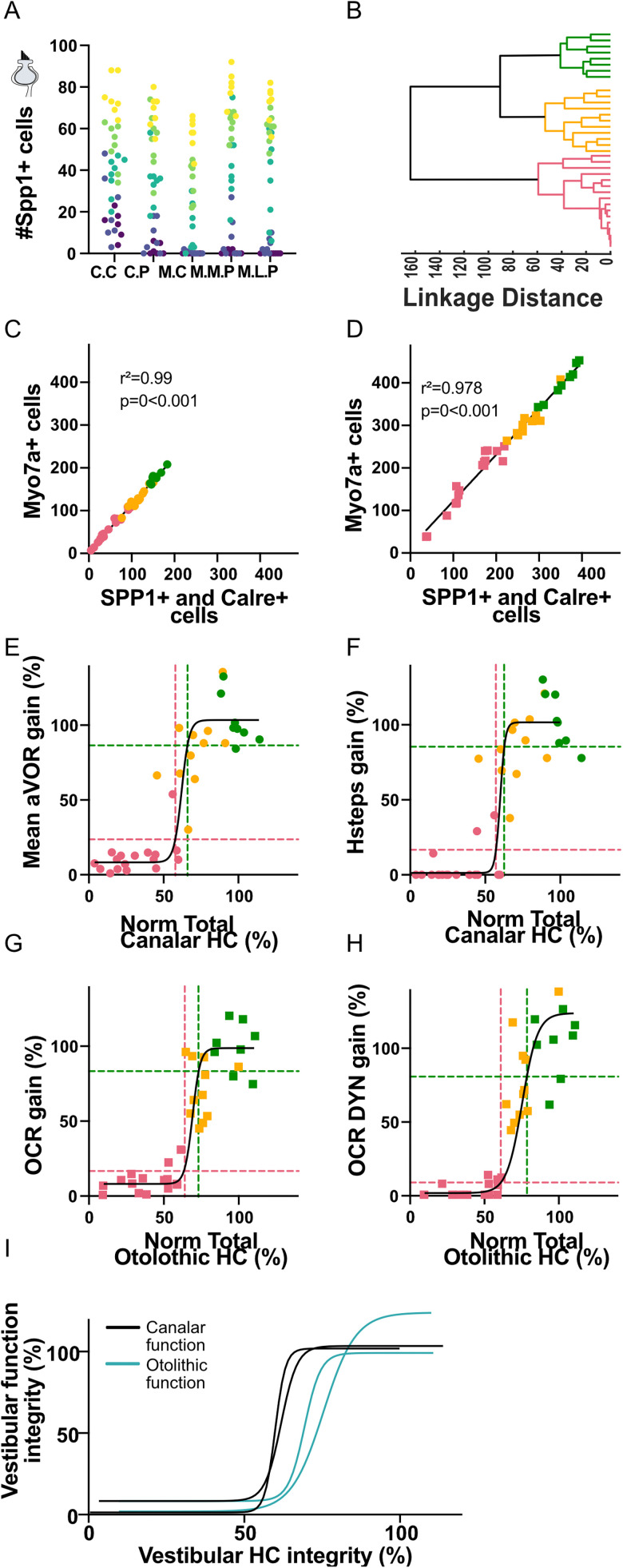
***A***, Number of type I hair cells counted in all regions of ampulla and macula (CC, crista central; CP, crista periphery; MC, macula central; MMP, macula medial periphery; MLP, macula lateral periphery). ***B***, Hierarchical clustering dendrogram of *n* = 36 mice based on five variables (type I HC number in all regions of both organs). Distance, Euclidean; Linkage, Complete. Colors indicate *k* = 3 clusters obtained by cutting at height 80. ***C***, ***D***, Number of Myo7a-labeled hair cells as a function of the additive number of Spp1- and Calre-labeled hair cells in the ampulla (***C***) and macula (***D***). ***E***, ***F***, Normalized mean aVOR gain (***E***) and Hsteps gain max (***F***) as a function of the normalized number of Myo7a-labeled hair cells in all regions of the ampulla. ***G***, ***H***, Normalized static OCR gain (***G***) and Dynamical OCR gain (***H***) as a function of the normalized number of Myo7a-labeled hair cells in all regions of the macula. In panel ***E–G*** the lowest (highest) threshold is obtained as the value of the sigmoidal fit, black line, for value of mean ± standard deviation of the pink (green) cluster group. ***I***, Superimposed 2D nonlinear fits of the vestibular hair cells integrity (%) as a function of the percentage of canal (black) and otolithic function (blue).

To determine these thresholds, we first grouped the mice based on their loss of type I HC observed in the different organs. As shown in Figure 4*A*, there is a large intragroup variability and intergroup overlap in the amount of HCI left in all regions, leading to an apparent continuum of type I HC integrity that ranges from the control animals (yellow) to the highest dose of the treatment ([40], dark purple). However, a cluster analysis on this dataset revealed three distinct categories of animals characterized by high (green), intermediate (orange), or low (pink) type I HC population integrity (Fig. 4*B*). We then validated that Myo7a is a pan-hair cell marker which in both canals (Fig. 4*C*) and otoliths (Fig. 4*D*) closely matches the number of both type I and type II HC. The high correlation between the addition of type I + type II and myo7a-labeled cells (canals *r*^2^ = 0.99, *p* < 0.0001, Fig. 4*C*; otoliths *r*^2^ = 0.979, *p* < 0.0001, Fig. 4*D*) is maintained for all three clusters, demonstrating that despite the ototoxic alteration of the HC, this marker remains an accurate index of total HC integrity in all our experimental conditions.

In Figure 4*E–H*, a comprehensive analysis of the global structure–function relationship encompassing all the hair cells was conducted by applying to the total hair cell (HC) residual number the same sigmoidal fitting procedure employed for the specific HC types and locations (Fig. 3). For each test, we defined the minimal function thresholds as the “mean + SD” gain of the low response cluster (pink dotted lines), while the normal vestibular function threshold was defined as the “mean − SD” gain of the high response cluster (green dotted lines). Using this methodology, normal thresholds represented >80% of function, while minimal threshold represents <20% of function (ordinate of the horizontal dotted line on panels in Fig. 4*E–H*).

The corresponding HC thresholds were then measured as the intercept between the function thresholds and the sigmoid curve. The quantity of HC to preserve minimal horizontal canal function was calculated at 57.8% for aVOR (Fig. 4*E*) and 57.03% for Hsteps (Fig. 4*F*), while the threshold for normal functions were calculated at 65.9 and 62.5%, respectively. Minimal HC thresholds for otolithic-dependent responses were slightly higher with lower bounds calculated at 63.5 and 60.9%, respectively, for static OCR (Fig. 4*G*) and Dynamic OCR (Fig. 4*H*). The HC thresholds for normal otolithic function were calculated at 73.1 and 78.5%, respectively, slightly higher than for canal function. This analysis suggests that canal-dependent function tested in our protocol remains in normal range when roughly two-thirds of HC are preserved but requires approximately one-half of HC to preserve minimal function. On the other hand, otolith-dependent function tested in our protocol requires approximately two-thirds of HC to maintain minimal function and three-fourths of HC to remain in normal range.

To further highlight the relation between the total number of hair cells in ampulla and macula and organ-specific function, the nonlinear fits of the normalized vestibular function presented in Figure 4*E–H* are superimposed on Figure 4*I*. This summary scheme illustrates that despite the experimental variability in measurements, interindividual variability, and differences in the parameters tested (static vs dynamic; long-lasting vs instantaneous responses), all vestibulo-ocular reflexes show a rather comparable dependency on the HC population integrity necessary to drive the eye movements, with no response observed when 50% of hair cell are lost, intermediate response when HC population is in range 50–80%, and normal function for both organs when HC integrity is >80%. A caveat is that the immunolabeled HCs might not all correspond to HCs that retain completely normal synaptic transmission, also there is evidence supporting this assumption. These boundaries therefore constitute conservative estimation regarding the quantity of hair cells which should be restored or preserved to ensure efficient therapy. We further discuss this point and other functional considerations below.

## Discussion

This study aimed to determine the minimal proportion of surviving vestibular HC necessary to maintain normal vestibular function, specifically assessed via the vestibulo-ocular reflex. Our experimentation involved exposing mice to increasing doses of an ototoxic compound, IDPN (3,3′-iminodipropionitrile), and revealed that vestibular function declines progressively, until vestibular responses are completely abolished. By combining immunohistochemistry with video-oculography in individuals, we found that VOR performance remains physiologically normal as long as at least 80% of type I HCs are preserved. We further found that no VOR response was detectable when fewer than 50% of type I HCs were preserved. To our knowledge, these thresholds represent the first quantitative benchmarks linking cellular survival to a quantifiable behavioral output of vestibular function, with relevance across species and potential translatability to clinical applications in humans.

### A single injection of IDPN at different doses is able to induce graded HC loss

We recently demonstrated (Schenberg et al., 2023) that exposure to IDPN at low doses in drinking water could cause a progressive and reversible alteration of type I HCs resulting in VOR dysfunction followed by recovery. This subchronic exposure protocol revealed that type I HCs are essential for maintaining VOR function. However, that previous approach did not allow for a quantitative assessment of the level of vestibular HC integrity required for proper VOR function.

In the present work, we used single IDPN injections at various concentration to induce a permanent loss of HCs. Although the ultimate mechanism of action of this chemical has yet to be identified, it has been shown to have selective toxicity on the HCs (Llorens et al., 1993; Llorens and Demêmes, 1994; Soler-Martín et al., 2006), including cochlear HCs (Crofton et al., 1994). Here, matching prior studies (Llorens et al., 1993; Soler-Martín et al., 2006; Martins-Lopes et al., 2019; Zeng et al., 2020; Maroto et al., 2021), a dose-dependent and graded vestibular HCs loss was achieved as evidenced by the quantification performed with the pan-HC marker Myo7a. One remaining question is whether the surviving HCs retain normal function, an issue not addressed in the present study and for which no direct evidence is provided. It is thus possible that the number of fully functional HCs is in fact lower than the number of HCs determined by Myo7a labeling. Nevertheless, the ototoxic effect of acute IDPN is time limited and a large amount of morphological data indicate that most of the HCs remaining after the degeneration process is complete retain normal structural features. This includes normal hair bundle morphology (Llorens et al., 1993; Llorens and Demêmes, 1994; Soler-Martín et al., 2006; Martins-Lopes et al., 2019), normal cell ultrastructure along with normal afferent and efferent endings ultrastructure (Llorens and Demêmes, 1994), normal calyceal junctions, as revealed by CASPR1 and tenascin-C labels and unaltered numbers of synaptic puncta in HCII as revealed by ribeye and PSD-95 labels (Maroto et al., 2021). We acknowledge that additional physiological data would be required to further substantiate this evidence, but as already mention, consider that these boundaries constitute a first conservative estimate of the structure–function relation for the preservation of the vestibulo-ocular pathway.

We validated the dynamics of functional loss in animals after a single injection at 24 mM (*n* = 3) and 40 mM (*n* = 2; data not shown). VOR decline was partial within the first 4 d postinjection, reaching complete loss after 1 week. To confirm the stability of our measurements, all animals of this study were tested at 2 and 3 weeks postinjection, confirming that VOR responses remained stable between these time points (Extended Data Fig. 1-1). While other ototoxic drugs such as cisplatin have been shown to induce a plateau in hair cell loss even at high doses (Ding et al., 2018), IDPN represents a cost-effective and practical pharmacological tool for inducing a wide range of permanent vestibular HC loss in mice, from minimal to complete (Llorens et al., 1993; Soler-Martín et al., 2006; Maroto et al., 2021).

### Quantification of the differential HC loss in subregions of ampulla and macula

It has long been recognized that the HCs found in the different regions of the vestibular organs differ by several characteristics (Goldberg, 2000; Eatock and Songer, 2011). Type I HCs and their associated calyx-shaped synapse were originally thought to be more numerous in the apex/center of the cristae ampullaris and in the central striola region of the maculae of the otolith organs (Eatock and Songer, 2011), with type II HCs more prevalent in peripheral and nonstriolar zones of the different vestibular organs, which are also characterized by a higher density of regular afferents (Goldberg, 2000). In our study, although we observed a higher number of type I HC compared with type II HC in the central regions, peripheral regions still showed a substantial number of type I HC.

Here, we conducted organ-specific vestibular tests in order to specifically assess the stabilizing eye movements that depend on the semicircular canals or otolith organs. As illustrated in Figure 2*E*, there was, however, a strong correlation between the HC loss observed in both set of organs, as well as between the different subregions. Type I HCs were lost in a dose-dependent fashion, and the loss was found to affect all organs and subregions uniformly. As such, our results do not allow to distinguish between the specific roles played by the type I HCs located in central versus peripheral areas of the organs. Similarly, type II HCs were mostly unaffected by IDPN, except at the highest dose tested. Indeed, in some individuals of the [32] and [40] groups, there was a moderate, nonsignificant, loss in the periphery of the organs. However, we note that these are the areas where the type II HCs are more abundant (Fig. 2*D*) and therefore, where the subtler effects of IDPN would be most readily detected by our quantification. Consequently, the main point that this dataset allows us to investigate lies in the role of type I and type II HCs in the encoding of head movements. As previously mentioned, type I and II vestibular HCs, through calyce, bouton, or dimorphic afferent terminals, are hypothesized to form complementary sensory channels, enabling the brain to encode distinct features of head position and motion in space.

The type I HCs and their associated calyceal synapse, with both quantal and nonquantal modes of transmission, appear particularly well suited for rapid information processing, likely essential for short-latency reflexive vestibular pathways. Thus, the encoding of rapid head movements with high-frequency content, whether generated during natural head movements (Carriot et al., 2017), bone-conducted vibrations (Curthoys et al., 2017), or jerk stimuli used to evoke vestibular sensory evoked potential (VsEPs; Jones et al., 2011), is thought to depend predominantly on type I HCs. In contrast, type II HCs with their button-type synaptic endings are hypothesized to be better suited for encoding low dynamic, tonic, and/or sustained stimuli, such as static head tilts. However, it is well recognized that most head movements simultaneously activate all type of HCs (Eatock, 2018) and that most afferents receive inputs from neighboring type I and type II HC (Lysakowski and Goldberg, 1997; Goldberg, 2000).

In our study, the loss of type I, but not type II, HCs led to a marked reduction in responses across all VOR tests. The presence of type I HCs is therefore shown to be critical for encoding all types of head movements, ranging from static head tilt to horizontal rotation with low to moderate dynamics. During static head tilt, head position relative to gravity is encoded as a change in the tonic firing rate of afferents (Goldberg and Fernandez, 1975), with better discrimination from regular than irregular afferents (Jamali et al., 2016). While this kind of stimulus with slow, sustained movement is usually thought to depend on the tonic neural elements (i.e., type II HC and regular afferents), our findings indicate that the type II HCs, located in both the central and peripheral parts of the otoliths and largely unaffected by the treatment, are insufficient on their own to mediate otolith-driven eye movements.

Overall, these observations confirm the critical role of type I HCs in the encoding of VOR, demonstrate that type II HCs alone are insufficient to drive compensatory eye movements, and suggest that both hair cell types are required to appropriately drive the discharge of vestibular afferents.

### Correlation of function with HC integrity indicate minimal and normal thresholds

By correlating structural and functional measures in individual subjects, we found that restoring at least 50% of overall HC integrity is likely required to support minimal vestibular function, while ∼80% appears necessary to achieve near-normal responses. Importantly, these thresholds reflect the total HC population and not specifically type I cells, although our findings suggest that type I HCs are especially critical for robust responses. In our protocol, VOR performance reflects contributions from both type II HC and the surviving population of type I hair cells, making it difficult to disentangle the specific role of each subtype in driving compensatory eye movements. When considering type I cells in isolation, thresholds for minimal and near-normal responses would be lower—approximately 40 and 60%, respectively. As such, our current estimates may overstate the actual proportion of type I HC required. Further studies will be needed to determine whether similar functional outcomes can be achieved with a smaller number of HCs, provided that a greater proportion of type I HCs is preserved.

In addition, by using tests specifically designed to differentiate canal- versus otolith-driven eye movements, we observed differences in the functional thresholds derived from both sets of organs (Fig. 4). These differences could be attributed to experimental limitations. For instance, the number of measurements acquired for each test differed, with fewer trials conducted for the otolithic tests compared with the canal-based ones, potentially affecting the resolution of the functional assessments. Moreover, the higher number of hair cells present in the otolithic organs relative to the semicircular canals could introduce a quantification bias.

Alternatively, this difference may reflect distinct population-coding strategies between the semicircular canals and the otolithic organs. In the ampullae, all HCs are activated by the same directional acceleration—i.e., based on head movements relative to the orientation of the main axis of the semicircular canal. In contrast, the maculae exhibit a more heterogeneous organization, with hair cells oriented along varying axes across different subregions. As a result, encoding a specific directional stimulus in the otolith organs may require a larger and more spatially distributed population of hair cells to achieve sufficient signal resolution (Simon et al., 2021).

### Restorative thresholds for vestibular recovery in gene therapy

Recent advancements in gene therapy, including the use of adeno-associated virus vectors (Isgrig et al., 2017), antisense oligonucleotides (Lentz et al., 2013), or even lentiviral vectors (Schott et al., 2023), have shown promise in restoring vestibular HCs and their associated functions. Our findings offer valuable quantitative benchmarks to guide the development and evaluation of such therapeutic strategies.

The minimal and near-normal thresholds of vestibular function identified at 60 and 80%, respectively, align with results from recent gene therapy investigations employing comparable methodologies. For instance, Schlecker et al. (2011) demonstrated that balance impairments induced by IDPN exposure could be reversed following adenoviral-mediated gene delivery, which resulted in near-complete restoration of utricular HCs, including type I HCs. Similarly, in a study by Jáuregui et al. (2024), retention of ∼50% of type I HCs after diphtheria toxin-induced damage was associated with significant preservation of vestibular function. When diphtheria toxin was also utilized to selectively target only HCs and no other vestibular cells, the regeneration of both type I and II otolithic HCs, ensured via Wnt pathway activation, achieved up to 58% of the original HC population (Lahlou et al., 2024b). However, the majority of the regenerated cells exhibited morphological and molecular features characteristic of type II HCs, with relatively few type I HCs restored. As a consequence, only partial functional recovery was observed, with modest improvements limited to the higher frequencies of the rotational VOR (Lahlou et al., 2024b). In contrast, regeneration rates below 50%—with utricular and canalar HC counts restored to only 21 and 14%, respectively—were insufficient to yield significant improvement in vestibulo-ocular reflexes (Jáuregui et al., 2024). These findings, taken together with our data, suggest that effective gene-based or pharmacologically based therapies for vestibular dysfunction should aim to restore at least 50–60% of the vestibular HC population to produce meaningful functional recovery. Notably, achieving higher proportions of regenerated type I HCs may be particularly important to support full restoration of reflexive vestibular responses.

A major limitation of current therapeutic strategies lies in their predominant emphasis on promoting the recovery of type II HC, resulting in either absent or suboptimal recovery of vestibular function (Jáuregui et al., 2024; Lahlou et al., 2024b). In neonatal mice, Lgr5-expressing supporting cells facilitate the regeneration of both type I and II HCs, enabling more complete restoration of vestibular HCs (Wang et al., 2015). In contrast, spontaneous regeneration of HCs in adult mice is limited and occurs primarily in response to injury. Notably, the newly regenerated vestibular cells exhibit a limited range of HC subtypes as regenerated cells have not been reported to include type I-like HCs (González-Garrido et al., 2021). Most newly formed cells display features characteristic of type II HCs, while others remain in immature or undifferentiated states (Kawamoto et al., 2009; Hicks et al., 2020; González-Garrido et al., 2021). This bias toward type II HC regeneration is also reflected in the preferential re-establishment of bouton-type afferent synapses, as opposed to calyx afferents that are associated with type I HCs (Zakir and Dickman, 2006). Despite this, pharmacological interventions have shown that both regular and irregular afferent activities can be reinstated following HC loss (Lahlou et al., 2024a). Together, these findings underscore a critical need for future therapeutic approaches to not only enhance HC regeneration but also ensure the recovery and functional integration of both type I and type II vestibular HCs. This dual focus will be essential to achieve optimal and physiologically meaningful restoration of vestibular function.

### Perspectives and conclusion

In our study, vestibular function was assessed through measurements of the vestibulo-ocular reflex (VOR), a well-characterized and quantifiable output of vestibular processing. However, the vestibular system contributes to a broad array of reflexive and cognitive functions beyond the VOR, including vestibulo-spinal reflexes, postural control, and higher-order processes such as spatial orientation and navigation (Cullen and Taube, 2017). Whether the functional thresholds we identified are generalizable to these other vestibular functions remains an open question. For example, vestibulo-spinal reflexes, which underlie balance and gait stability, may exhibit different sensitivity to hair cell loss, potentially requiring distinct levels of cellular preservation for functional maintenance. Similarly, cognitive processes that rely on vestibular input but integrate multisensory information may show greater resilience to partial vestibular damage due to substitution mechanisms. Moreover, it is important to consider that vestibular functional preservation may not be determined solely by the quantity of surviving hair cells. The integrity of synaptic connections with vestibular afferents and the plasticity of central pathways (Beraneck and Idoux, 2012), particularly within the brainstem, were reported to play key roles in shaping residual function and recovery potential.

In summary, our findings emphasize the pivotal role of type I HCs in maintaining vestibular function and validate IDPN-induced HC loss as a robust model for investigating vestibular decline. Furthermore, the quantitative thresholds identified in this study offer valuable benchmarks for guiding future regenerative therapies aimed at restoring vestibular function and underscore the need for comprehensive assessments that extend beyond the VOR to fully capture the complexity of vestibular system recovery.

## Synthesis

Reviewing Editor: Nicholas Priebe, The University of Texas at Austin Decisions are customarily a result of the Reviewing Editor and the peer reviewers coming together and discussing their recommendations until a consensus is reached. When revisions are invited, a fact-based synthesis statement explaining their decision and outlining what is needed to prepare a revision will be listed below. The following reviewer(s) agreed to reveal their identity: Mark Warchol. Note: If this manuscript was transferred from JNeurosci and a decision was made to accept the manuscript without peer review, a synthesis may not be available.

Two reviewers evaluated the manuscript "Hair cell population integrity necessary to preserve vestibular function." The manuscript addresses how many hair cells, and which type, are needed to support vestibular function. Both reviewers recognized that the answers are important in inter-connected fundamental and translational ways. The data presented show that decreased vestibular function is correlated with hair cell loss. The loss of ~50% of the normal hair cell population may be sufficient to cause serious deficits in vestibular reflexes. In particular, the authors demonstrate a strong correlation between type I hair cell loss and reflex deficits. Both reviewers agreed that more information on the health of the calyx terminals following ototoxicity would significantly strengthen the manuscript. The reviewers were very positive in their evaluations, but raised a few questions, detailed below, which should be useful in revising your manuscript, if you decide to resubmit it to eNeuro or to another journal.

Major Issues:

1. Clarifications of methods employed

The manuscript needs a clearer description of how hair cells were counted. How were areas for counting chosen in each tissue? How were they standardized across animals? What is the "global" number? There is an implication that the whole epithelium was analyzed (lines 715-16) but the numbers are <10% of the totals (whether for rat epithelia in Borrajo et al. 2024 or for mouse utricle, Li et al. 2021) - in which case it is important to describe the ROI sampled.

Likewise there is no in-text description of how zones were recognized and how samples were standardized across animals. The insets in Fig. 2E (top) suggest approximate locations of the histological counts - though this needs to made more clear. In the insets it is not possible to tell if the "MLP" slice crosses the LPR and therefore includes both "lateral" and "medial extrastriola" as used in other descriptions (see Doris Wu recent papers). Striola, lateral and medial extrastriola have real meaning in terms of genetic origins and relation to landmarks such as the LPR (Ono ... Wu, 2020 and Ono...Tarchini, 2024), and the original morphophysiological papers of Goldberg and colleagues. The results in this paper will be more widely valuable if epithelial locations reference directly the literature on zones in rodent epithelia.

2. The impact of ototoxicity

It is assumed hair cells that survive after IDPN ototoxicity retain normal function. This may be true, but given that we have very minimal knowledge of how IDPN affects (and kills) hair cells, it is recommended that the authors acknowledge this point. It is possible that some of the surviving hair cells have compromised function.

It is unfortunate that only hair cells (types I and II) were immunolabeled, without also labeling and quantifying the numbers of calyx nerve terminals. Low doses of IDPN have been shown to cause loss of calyx terminals, without accompanying loss of type I hair cells (e.g., Greguske et al., Arch Toxicol, 2019). In the present study, is it possible to rule out any deficits that might have resulted from calyx loss vs. hair cell loss?

A similar issue also applies to type II hair cells. Is it possible that IDPN can cause synaptopathy of bouton terminals, without killing type II's? This would also complicate the conclusions of the study.

Some quantification of calretinin-labeled calyces (and associated myosin VIIa-labeled hair cells) would be an excellent addition to the manuscript.

3. Presentation of the vestibular testing data

Only summary plots (X-Y graphs) are provided. The impact of the manuscript would be enhanced by including exemplars, as provided in Fig. 1 in Schoenberg et al., eLife, 2023 - especially for reaching beyond the immediate sub-field.

4. Statistical issues

Indicate in Methods whether you tested for homogeneity of variance and whether the data formed ~normal distributions (as required for use of parametric ANOVA).

Consider including 1) effect size (Cohen's d) for significant results; and 2) the power (post-hoc estimate) of NS results - while the latter is not a perfect tool, it helps to convey the chance that results appear "NS" because they are of low power.

For the cluster analysis (Fig. 4B), provide details about the methods - which software and clustering algorithm? Labels and Fig. Legend should include more information - without more information, the value of the cluster analysis is not clear.

Minor Issues:

By convention, there is a second period on "i.e.".

Lines 111-113: While it is correct that type II hair cells are associated with regularly discharging afferents in the extrastriolar regions of the utricle, the association is somewhat complex. Most type II's provide information to calyces via dimorphic terminals. These terminals probably summate synaptic input from a type I hair cell, as well as numerous type II hair cells. There is another population of afferents (known as 'bouton afferents') that receive input exclusively from type II's, but those are a minority of the afferent population (<15%). It is difficult to separate the contribution of type I I hair cells to dimorphic afferents (which also contact type I's) from their role in driving activity in bouton afferents.

Lines 132-141: It is probably worth noting that the limited regeneration that does occur in the mammalian vestibular organs does not result in any functional recovery. (Reference:

Jáuregui EJ, Scheinman KL, Bibriesca Mejia IK, Pruett L, Zaini H, Finkbeiner

C, Phillips JA, Gantz JA, Nguyen TB, Phillips JO, Stone JS. Sensorineural

correlates of failed functional recovery after natural regeneration of

vestibular hair cells in adult mice. Front Neurol. 2024 Mar 8;15:1322647. doi:

10.3389/fneur.2024.1322647).

Line 180: I believe that Bode should be capitalized (Bode plots were first used by Hendrik Bode).

Lines 214-230 and Figure 2: The details of hair cell quantification are not clear. Did the authors count all hair cells in the given regions? What are the units of the y axis of the plots in 2B? (There values are consistent with being hair cell densities, but this is not specified.). Since an accurate assessment of surviving hair cells is central to the conclusions of this study, it is essential that the quantification methods be fully described and that hair numbers are clearly reported.

Lines 247-279: A similar issue affects my ability to interpret these plots - Since it is not clear how hair cells were quantified, it is not possible to fully understand these plots. There appears to be a strong correlation between hair cell number and vestibular function.

Lines 281-325: These paragraphs outline a rather complex analysis that ultimately concludes that suggests that loss of >50% of the total hair cell population results in the elimination of vestibular function. As noted above, this assertion is based on the assumption that all SPP-labeled hair cells (type I's) retain their calyx terminals and that neurotransmission among all remaining hair cells is functioning normally. Are those assumptions justifiable?

Line 592: "utricle" not "utricule"

Line 626: "Materiels et Methods"

## References

[B1] Agrawal Y, Ward BK, Minor LB (2013) Vestibular dysfunction: prevalence, impact and need for targeted treatment Chabbert C, ed. J Vestib Res 23:113–117. 10.3233/VES-130498 24177344 PMC4069154

[B2] Angelaki DE, Cullen KE (2008) Vestibular system: the many facets of a multimodal sense. Annu Rev Neurosci 31:125–150. 10.1146/annurev.neuro.31.060407.125555 18338968

[B4] Beraneck M, Idoux E (2012) Reconsidering the role of neuronal intrinsic properties and neuromodulation in vestibular homeostasis. Front Neurol 3:3–25. 10.3389/fneur.2012.00025 22403570 PMC3289128

[B3] Beraneck M, Bojados M, Le Séac’h A, Jamon M, Vidal P-P (2012) Ontogeny of mouse vestibulo-ocular reflex following genetic or environmental alteration of gravity sensing (Gilestro GF, ed). PLoS One 7:e40414. 10.1371/journal.pone.0040414 22808156 PMC3393735

[B5] Borrajo M, Sedano D, Palou A, Giménez-Esbrí V, Barrallo-Gimeno A, Llorens J (2024) Maturation of type I and type II rat vestibular hair cells in vivo and in vitro. Front Cell Dev Biol 12:1404894. 10.3389/fcell.2024.1404894 38895157 PMC11183282

[B6] Carcaud J, França de Barros F, Idoux E, Eugène D, Reveret L, Moore LE, Vidal P-P, Beraneck M (2017) Long-lasting visuo-vestibular mismatch in freely-behaving mice reduces the vestibulo-ocular reflex and leads to neural changes in the direct vestibular pathway. eNeuro 4:ENEURO.0290-16.2017. 10.1523/ENEURO.0290-16.2017 PMC5354632 28303261

[B7] Carriot J, Jamali M, Chacron MJ, Cullen KE (2017) The statistics of the vestibular input experienced during natural self-motion differ between rodents and primates: natural vestibular input in rodents and monkeys. J Physiol 595:2751–2766. 10.1113/JP273734 28083981 PMC5390882

[B8] Contini D, Holstein GR, Art JJ (2024) Simultaneous recordings from vestibular type I hair cells and their calyceal afferents in mice. Front Neurol 15:1434026. 10.3389/fneur.2024.1434026 39263277 PMC11387672

[B9] Crofton KM, Lassiter TL, Rebert CS (1994) Solvent-induced ototoxicity in rats: an atypical selective mid-frequency hearing deficit. Hear Res 80:25–30. 10.1016/0378-5955(94)90005-1 7852200

[B10] Cullen KE, Taube JS (2017) Our sense of direction: progress, controversies and challenges. Nat Neurosci 20:1465. 10.1038/nn.4658 29073639 PMC10278035

[B11] Curthoys IS, MacDougall HG, Vidal P-P, de Waele C (2017) Sustained and transient vestibular systems: a physiological basis for interpreting vestibular function. Front Neurol 8:117. 10.3389/fneur.2017.00117 28424655 PMC5371610

[B12] Dechesne CJ, Winsky L, Kim HN, Goping G, Vu TD, Wenthold RJ, Jacobowitz DM (1991) Identification and ultrastructural localization of a calretinin-like calcium-binding protein (protein 10) in the guinea pig and rat inner ear. Brain Res 560:139–148. 10.1016/0006-8993(91)91224-O 1722130

[B13] Ding D, Jiang H, Zhang J, Xu X, Qi W, Shi H, Yin S, Salvi R (2018) Cisplatin-induced vestibular hair cell lesion-less damage at high doses. J Otol 13:115–121. 10.1016/j.joto.2018.08.002 30671086 PMC6335437

[B14] Eatock RA (2018) Specializations for fast signaling in the amniote vestibular inner ear. Integr Comp Biol 58:341–350. 10.1093/icb/icy069 29920589 PMC6104706

[B15] Eatock RA, Songer JE (2011) Vestibular hair cells and afferents: two channels for head motion signals. Annu Rev Neurosci 34:501–534. 10.1146/annurev-neuro-061010-113710 21469959

[B16] Elliott KL, Fritzsch B, Duncan JS (2018) Evolutionary and developmental biology provide insights into the regeneration of organ of corti hair cells. Front Cell Neurosci 12:252. 10.3389/fncel.2018.00252 30135646 PMC6092489

[B17] Emptoz A, et al. (2017) Local gene therapy durably restores vestibular function in a mouse model of Usher syndrome type 1G. Proc Natl Acad Sci U S A 114:9695–9700. 10.1073/pnas.1708894114 28835534 PMC5594693

[B18] Fleihan T, Nader ME, Dickman JD (2024) Cisplatin vestibulotoxicity: a current review. Front Surg 11:1437468. 10.3389/fsurg.2024.1437468 39421409 PMC11484025

[B19] França de Barros F, Schenberg L, Tagliabue M, Beraneck M (2020) Long term visuo-vestibular mismatch in freely behaving mice differentially affects gaze stabilizing reflexes. Sci Rep 10:20018. 10.1038/s41598-020-77026-w 33208812 PMC7674424

[B20] Goldberg JM (2000) Afferent diversity and the organization of central vestibular pathways. Exp Brain Res 130:277–297. 10.1007/s002210050033 10706428 PMC3731078

[B21] Goldberg JM, Fernandez C (1975) Responses of peripheral vestibular neurons to angular and linear accelerations in the squirrel monkey. Acta Otolaryngol 80:101–110. 10.3109/00016487509121307 809987

[B22] Golub JS, Tong L, Ngyuen TB, Hume CR, Palmiter RD, Rubel EW, Stone JS (2012) Hair cell replacement in adult mouse utricles after targeted ablation of hair cells with diphtheria toxin. J Neurosci 32:15093–15105. 10.1523/JNEUROSCI.1709-12.2012 23100430 PMC3544304

[B23] González-Garrido A, Pujol R, López-Ramírez O, Finkbeiner C, Eatock RA, Stone JS (2021) The differentiation status of hair cells that regenerate naturally in the vestibular inner ear of the adult mouse. J Neurosci 41:7779–7796. 10.1523/JNEUROSCI.3127-20.2021 34301830 PMC8445055

[B24] Halmagyi GM, Fattore CM, Curthoys IS, Wade S (1994) Gentamicin vestibulotoxicity. Otolaryngol Head Neck Surg 111:571–574. 10.1177/019459989411100506 7970794

[B25] Hasson T, Gillespie PG, Garcia JA, MacDonald RB, Zhao Y, Yee AG, Mooseker MS, Corey DP (1997) Unconventional myosins in inner-ear sensory epithelia. J Cell Biol 137:1287–1307. 10.1083/jcb.137.6.1287 9182663 PMC2132524

[B26] Hicks KL, Wisner SR, Cox BC, Stone JS (2020) Atoh1 is required in supporting cells for regeneration of vestibular hair cells in adult mice. Hear Res 385:107838. 10.1016/j.heares.2019.107838 31751832 PMC7153450

[B27] Hoffman LF, Choy KR, Sultemeier DR, Simmons DD (2018) Oncomodulin expression reveals new insights into the cellular organization of the murine utricle striola. JARO 19:33–51. 10.1007/s10162-017-0652-6 29318409 PMC5783930

[B28] Huterer M, Cullen KE (2002) Vestibuloocular reflex dynamics during high-frequency and high-acceleration rotations of the head on body in rhesus monkey. J Neurophysiol 88:13–28. 10.1152/jn.2002.88.1.13 12091529

[B29] Idoux E, Tagliabue M, Beraneck M (2018) No gain no pain: relations between vestibulo-ocular reflexes and motion sickness in mice. Front Neurol 9:918. 10.3389/fneur.2018.00918 30483206 PMC6240678

[B30] Isgrig K, et al. (2017) Gene therapy restores balance and auditory functions in a mouse model of Usher syndrome. Mol Ther 25:780–791. 10.1016/j.ymthe.2017.01.007 28254438 PMC5363211

[B31] Jamali M, Chacron MJ, Cullen KE (2016) Self-motion evokes precise spike timing in the primate vestibular system. Nat Commun 7:13229. 10.1038/ncomms13229 27786265 PMC5095295

[B32] Jáuregui E, et al. (2024) Sensorineural correlates of failed functional recovery after natural regeneration of vestibular hair cells in adult mice. Front Neurol 15:1322647. 10.3389/fneur.2024.1322647 38523617 PMC10960365

[B33] Ji YR, et al. (2022) Function of bidirectional sensitivity in the otolith organs established by transcription factor Emx2. Nat Commun 13:6330. 10.1038/s41467-022-33819-3 36280667 PMC9592604

[B34] Jones TA, Jones SM, Vijayakumar S, Brugeaud A, Bothwell M, Chabbert C (2011) The adequate stimulus for mammalian linear vestibular evoked potentials (VsEPs). Hear Res 280:133–140. 10.1016/j.heares.2011.05.005 21664446 PMC3826178

[B35] Kawamoto K, Izumikawa M, Beyer LA, Atkin GM, Raphael Y (2009) Spontaneous hair cell regeneration in the mouse utricle following gentamicin ototoxicity. Hear Res 247:17–26. 10.1016/j.heares.2008.08.010 18809482 PMC2905733

[B36] Lahlou G, et al. (2024a) Extended time frame for restoring inner ear function through gene therapy in Usher1G preclinical model. JCI Insight 9:e169504. 10.1172/jci.insight.169504 38194286 PMC10967394

[B37] Lahlou H, Zhu H, Zhou W, Edge ASB (2024b) Pharmacological regeneration of sensory hair cells restores afferent innervation and vestibular function. J Clin Invest 134:e181201. 10.1172/JCI181201 39316439 PMC11563683

[B38] Lentz JJ, Jodelka FM, Hinrich AJ, McCaffrey KE, Farris HE, Spalitta MJ, Bazan NG, Duelli DM, Rigo F, Hastings ML (2013) Rescue of hearing and vestibular function by antisense oligonucleotides in a mouse model of human deafness. Nat Med 19:345–350. 10.1038/nm.3106 23380860 PMC3657744

[B40] Llorens J, Demêmes D (1994) Hair cell degeneration resenting from 3,3’-imiodiprpiunitril toxicity in the rat vestibular epithelia. Hear Res 76:78–86. 10.1016/0378-5955(94)90090-6 7928719

[B39] Llorens J, Demêmes D, Sans A (1993) The behavioural syndrome caused by 3’3-iminodipropionitrile and related nitriles in the rat is associated with degeneration of the vestibular sensory hair cells. Toxicol Appl Pharmacol 132:199–210. 10.1006/taap.1993.1238 8248927

[B41] Lopez I, Ishiyama G, Tang Y, Tokita J, Baloh RW, Ishiyama A (2005) Regional estimates of hair cells and supporting cells in the human crista ampullaris. J Neurosci Res 82:421–431. 10.1002/jnr.20652 16211560

[B42] Lysakowski A, Goldberg JM (1997) A regional ultrastructural analysis of the cellular and synaptic architecture in the chinchilla cristae ampullares. J Comp Neurol 389:419–443. 10.1002/(SICI)1096-9861(19971222)389:3<419::AID-CNE5>3.0.CO;2-3 9414004 PMC11468975

[B43] Mackowetzky K, Yoon KH, Mackowetzky EJ, Waskiewicz AJ (2021) Development and evolution of the vestibular apparatuses of the inner ear. J Anat 239:801–828. 10.1111/joa.13459 34047378 PMC8450482

[B44] Maroto AF, Barrallo-Gimeno A, Llorens J (2021) Relationship between vestibular hair cell loss and deficits in two anti-gravity reflexes in the rat. Hear Res 410:108336. 10.1016/j.heares.2021.108336 34481267

[B45] Martins-Lopes V, Bellmunt A, Greguske EA, Maroto AF, Boadas-Vaello P, Llorens J (2019) Quantitative assessment of anti-gravity reflexes to evaluate vestibular dysfunction in rats. J Assoc Res Otolaryngol 20:553–563. 10.1007/s10162-019-00730-6 31297642 PMC6962417

[B46] Maudoux A, Vitry S, El-Amraoui A (2022) Vestibular deficits in deafness: clinical presentation, animal modeling, and treatment solutions. Front Neurol 13:816534. 10.3389/fneur.2022.816534 35444606 PMC9013928

[B47] McInturff S, Burns JC, Kelley MW (2018) Characterization of spatial and temporal development of type I and type II hair cells in the mouse utricle using new cell-type-specific markers. Biol Open 7:bio038083. 10.1242/bio.038083 30455179 PMC6262869

[B48] Merchant SN, Tsuji K, Wall C, Velázquez-Villaseñor L, Glynn RJ, Rauch SD (2000) Temporal bone studies of the human peripheral vestibular system. Ann Otol Rhinol Laryngol 109:3–13. 10.1177/00034894001090s502 10821229

[B49] Oommen BS, Stahl JS (2008) Eye orientation during static tilts and its relationship to spontaneous head pitch in the laboratory mouse. Brain Res 1193:57–66. 10.1016/j.brainres.2007.11.053 18178173 PMC2278379

[B50] Paplou V, Schubert NMA, Pyott SJ (2021) Age-related changes in the cochlea and vestibule: shared patterns and processes. Front Neurosci 15:680856. 10.3389/fnins.2021.680856 34539328 PMC8446668

[B51] Rauch SD, Velazquez-Villaseñor L, Dimitri PS, Merchant SN (2001) Decreasing hair cell counts in aging humans. Ann N Y Acad Sci 942:220–227. 10.1111/j.1749-6632.2001.tb03748.x 11710464

[B52] Rüsch A, Lysakowski A, Eatock RA (1998) Postnatal development of type I and type II hair cells in the mouse utricle: acquisition of voltage-gated conductances and differentiated morphology. J Neurosci 18:7487–7501. 10.1523/JNEUROSCI.18-18-07487.1998 9736667 PMC6793223

[B53] Sayyid ZN, Wang T, Chen L, Jones SM, Cheng AG (2019) Atoh1 directs regeneration and functional recovery of the mature mouse vestibular system. Cell Rep 28:312–324.e4. 10.1016/j.celrep.2019.06.028 31291569 PMC6659123

[B54] Schenberg L, Palou A, Simon F, Bonnard T, Barton C-E, Fricker D, Tagliabue M, Llorens J, Beraneck M (2023) Multisensory gaze stabilization in response to subchronic alteration of vestibular type I hair cells. Elife 12:RP88819. 10.7554/eLife.88819.3 38019267 PMC10686621

[B55] Schlecker C, Praetorius M, Brough DE, Presler RG, Hsu C, Plinkert PK, Staecker H (2011) Selective atonal gene delivery improves balance function in a mouse model of vestibular disease. Gene Ther 18:884–890. 10.1038/gt.2011.33 21472006 PMC3136627

[B56] Schott JW, Huang P, Morgan M, Nelson-Brantley J, Koehler A, Renslo B, Büning H, Warnecke A, Schambach A, Staecker H (2023) Third-generation lentiviral gene therapy rescues function in a mouse model of Usher 1B. Mol Ther 31:3502–3519. 10.1016/j.ymthe.2023.10.018 37915173 PMC10727968

[B57] Selimoğlu E, Kalkandelen S, Erdoğan F (2003) Comparative vestibulotoxicity of different aminoglycosides in the Guinea pigs. Yonsei Med J 44:517–522. 10.3349/ymj.2003.44.3.517 12833591

[B58] Simon F, Pericat D, Djian C, Fricker D, Denoyelle F, Beraneck M (2020) Surgical techniques and functional evaluation for vestibular lesions in the mouse: unilateral labyrinthectomy (UL) and unilateral vestibular neurectomy (UVN). J Neurol 267:51–61. 10.1007/s00415-020-09960-8 32556569 PMC7718198

[B59] Simon F, Tissir F, Michel V, Lahlou G, Deans M, Beraneck M (2021) Implication of vestibular hair cell loss of planar polarity for the canal and otolith-dependent vestibulo-ocular reflexes in Celsr1–/– mice. Front Neurosci 15:750596. 10.3389/fnins.2021.750596 34790090 PMC8591238

[B60] Soler-Martin C, Diez-Padrisa N, Boadas-Vaello P, Llorens J (2006) Behavioral disturbances and hair cell loss in the inner ear following nitrile exposure in mice, Guinea pigs, and frogs. Toxicol Sci 96:123–132. 10.1093/toxsci/kfl186 17159233

[B61] Songer JE, Eatock RA (2013) Tuning and timing in mammalian type I hair cells and calyceal synapses. J Neurosci 33:3706–3724. 10.1523/JNEUROSCI.4067-12.2013 23426697 PMC3857958

[B62] Stahl JS (2004) Using eye movements to assess brain function in mice. Vision Res 44:3401–3410. 10.1016/j.visres.2004.09.011 15536008

[B63] Walther LE, Westhofen M (2008) Presbyvertigo-aging of otoconia and vestibular sensory cells. J Vestib Res 17:89–92. 10.3233/VES-2007-172-303 18413901

[B64] Wang T, et al. (2015) Lgr5+ cells regenerate hair cells via proliferation and direct transdifferentiation in damaged neonatal mouse utricle. Nat Commun 6:6613. 10.1038/ncomms7613 25849379 PMC4391285

[B65] Wang T, Niwa M, Sayyid ZN, Hosseini DK, Pham N, Jones SM, Ricci AJ, Cheng AG (2019) Uncoordinated maturation of developing and regenerating postnatal mammalian vestibular hair cells. PLoS Biol 17:e3000326. 10.1371/journal.pbio.3000326 31260439 PMC6602158

[B66] Wu P, Wu X, Zhang C, Chen X, Huang Y, Li H (2021) Hair cell protection from ototoxic drugs. Neural Plast 2021:4909237. 10.1155/2021/4909237 34335732 PMC8289577

[B67] Zakir M, Dickman JD (2006) Regeneration of vestibular otolith afferents after ototoxic damage. J Neurosci 26:2881–2893. 10.1523/JNEUROSCI.3903-05.2006 16540565 PMC6673967

[B68] Zeng S, et al. (2020) Toxic effects of 3,3 ′-Iminodipropionitrile on vestibular system in adult C57BL/6J mice in vivo. Neural Plast 2020:1823454. 10.1155/2020/1823454 32714382 PMC7354661

