# Supplementary figures and images for: Sensory-Cell Population Integrity Required to Preserve Minimal and Normal Vestibulo-ocular Reflexes Reveals the Critical Role of Type I Hair Cells in Canal- and Otolith-Specific Functions
Source: eNeuro. 2026 Feb 19;13(2):ENEURO.0303-25.2026. doi: 10.1523/ENEURO.0303-25.2026 (PMC12928769; doi:10.1523/ENEURO.0303-25.2026)

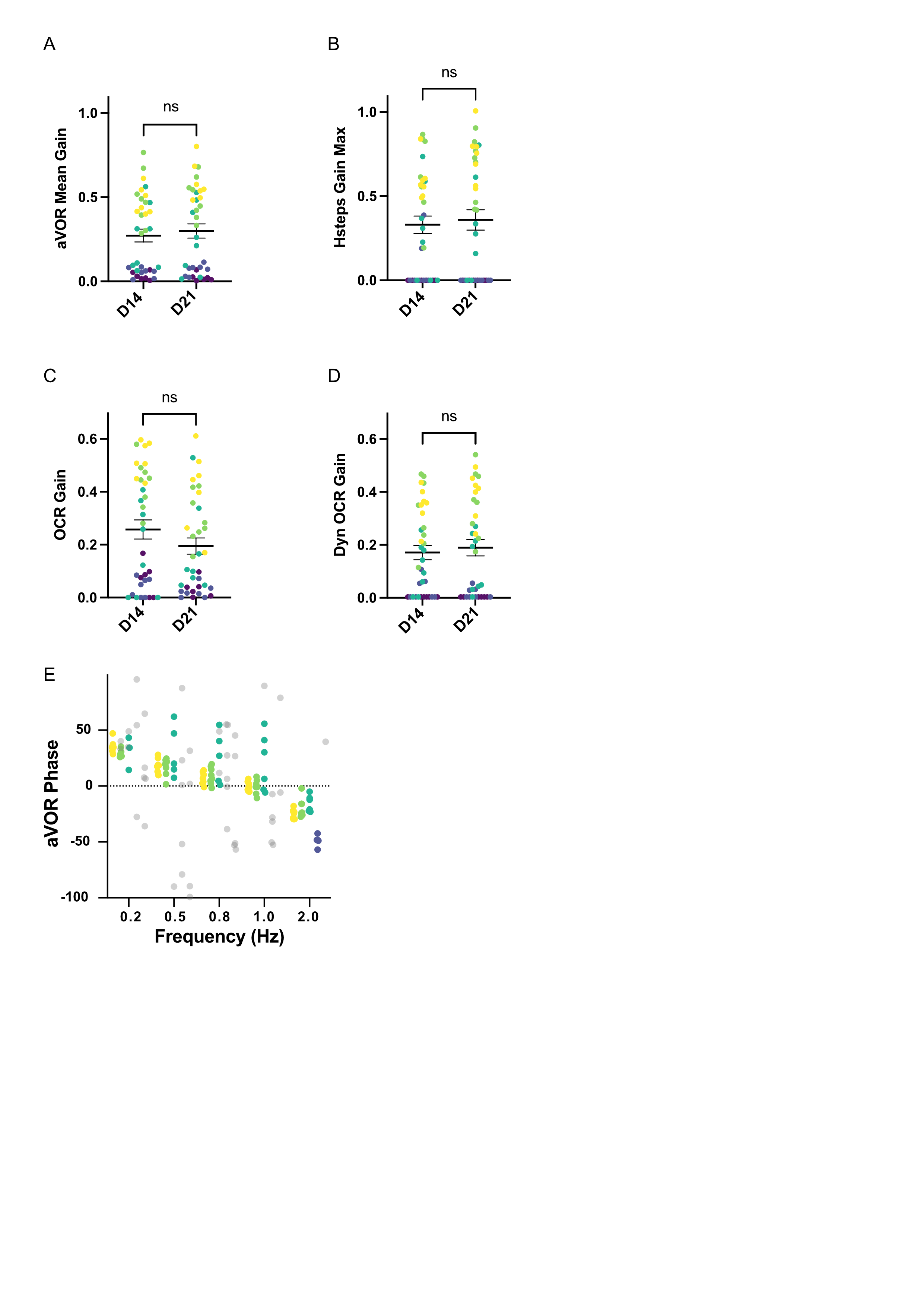

Supplement: Figure 1-1 — Mean aVOR gain (A), HStep gain (B), static OCR gain (C), Dynamic OCR gain (D) of all (n = 36) mice at D14 and D21 after injection. E) aVOR Phase values at different frequencies for the 5 different concentration tested. Abnormal phase values associated with gain<0.1 and VAF<0.5 are represented in grey. Sample size are [Control] (n = 7), [16] (n = 8), [24] (n = 8), [32] (n = 7) and [40] (n = 6). Download Figure 1-1, TIF file. [file eneuro-13-ENEURO.0303-25.2026-s002.tif]

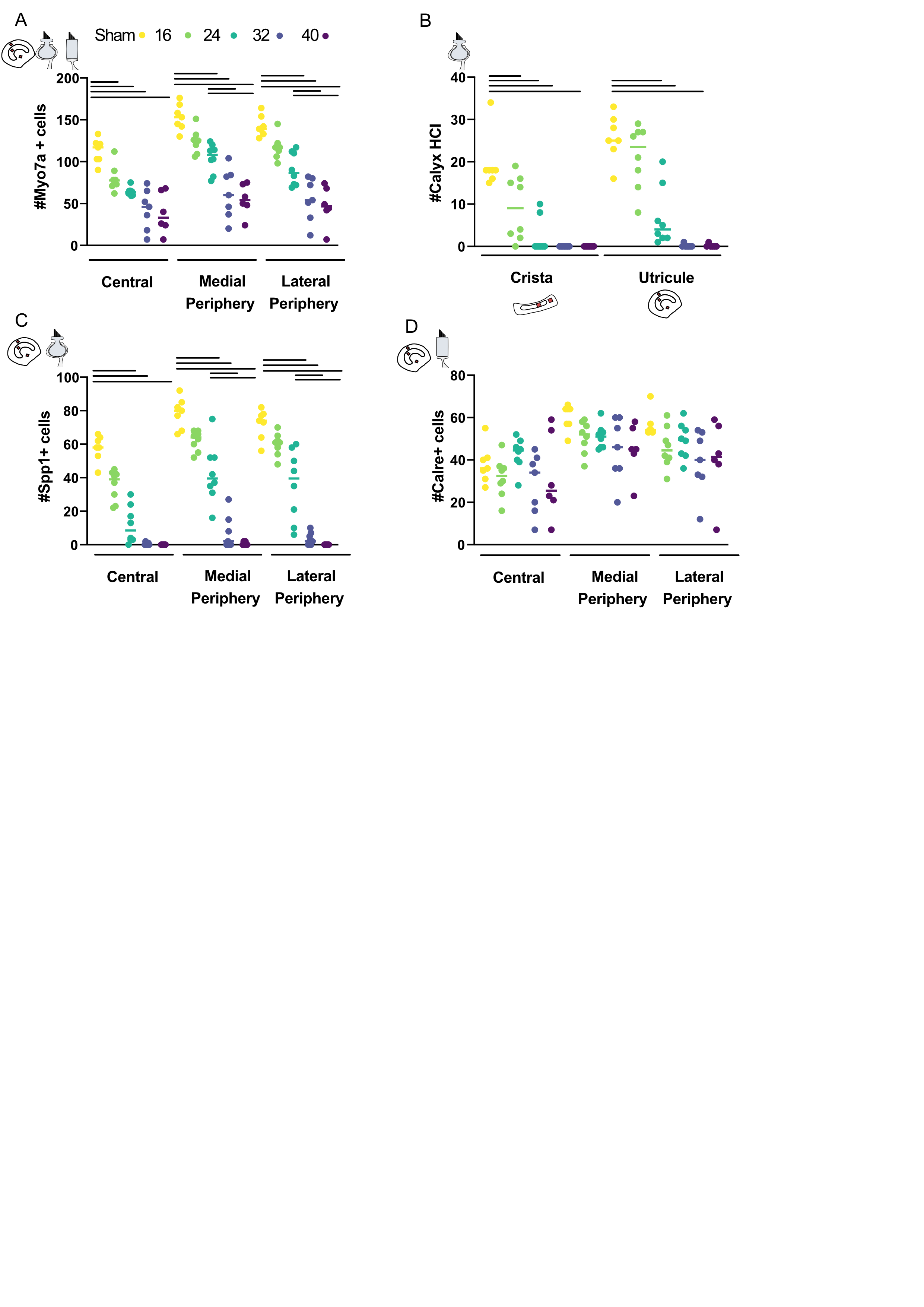

Supplement: Figure 2-1 — (A)Cell count of calyx-only type I HC in the crista and utricle. (B,C,D) Cell count of hair cells in the central and peripheral utricle for all IDPN doses, with (A) Myo7a + marker (all HC) (B) Spp1 + marker (type I specific marker), (C) Calre + marker (type II specific). Sample size Sham (n = 7), [16] (n = 8), [24] (n = 8), [32] (n = 7) and [40] (n = 6). Download Figure 2-1, TIF file. [file eneuro-13-ENEURO.0303-25.2026-s003.tif]
