# Supplementary material for: Sensory-Cell Population Integrity Required to Preserve Minimal and Normal Vestibulo-ocular Reflexes Reveals the Critical Role of Type I Hair Cells in Canal- and Otolith-Specific Functions
Source: eNeuro. 2026 Feb 19;13(2):ENEURO.0303-25.2026. doi: 10.1523/ENEURO.0303-25.2026 (PMC12928769; doi:10.1523/ENEURO.0303-25.2026)
Supplement: Data 1 — Download Data 1, RTF file. [file eneuro-13-ENEURO.0303-25.2026-s001.rtf]

Organ_name={'CRISTA','UTRICLE'};Locations={'CRISTA - Center', 'CRISTA - Periphery', 'UTRICLE - Striola', 'UTRICLE - Periph Medial', 'UTRICLE - Periph Lateral'};%POUR VORparHC1_TailLift=optimalSigmoidFitting([HC1(:,1)],vor(:,1),Organ_name,['HC1_' Locations{1}],['vor' num2str(freqVor(1)) 'Hz'],['par' 'HC1_' Locations{1} 'vor_' num2str(freqVor(1)) 'Hz' '.mat']);%POUR OCR%parHC1_TailLift=optimalSigmoidFitting([HC1(:,3)],OVAR(:,1),Organ_name,['HC1_' Locations{3}],['OVAR_'],['par' 'HC1_' Locations{3} 'OVAR' '.mat']);function [parOut]=optimalSigmoidFitting(cellN,Y,Organ_name,cell_Name,bahav_name,matfilename)options = optimoptions('fmincon');options = optimset('Display','iter');%,'Algorithm','sqp'%options.MaxFunctionEvaluations =4*10^4;set(0,'units','pixels')Pix_SS = get(0,'screensize');figure('Name',['sigmoidInterp - ' cell_Name ' vs ' bahav_name],'Units', 'pixels','Position',[0 0 Pix_SS(3)-20 floor((Pix_SS(4)-105)/2)]);%figure('Name',['sigmoidInterp - ' cell_Name ' vs ' bahav_name],'Position',[0 floor((Pix_SS(4)-105)/2)+1 Pix_SS(3) floor((Pix_SS(4)-105)/2)]);for org=1:size(cellN,2) %organs    subplot(1,size(cellN,2),org);hold on    plot(cellN(:,org),Y,'o')    X=min(cellN(:,org)):max(cellN(:,org));            if ~exist(matfilename,"file")                %X0                 ymin    ymax    K        par0 = [mean(cellN(:,org))  min(Y) max(Y) (max(Y)-min(Y))/(max(cellN(:,org))-min(cellN(:,org)));...                median(cellN(:,org))  min(Y) max(Y) (max(Y)-min(Y))/(max(cellN(:,org))-min(cellN(:,org)));...                0                   min(Y) max(Y) (max(Y)-min(Y))/(max(cellN(:,org))-min(cellN(:,org)));...                100  min(Y) max(Y) (max(Y)-min(Y))/(max(cellN(:,org))-min(cellN(:,org)));...                mean(cellN(:,org))  min(Y) max(Y) 2;...                median(cellN(:,org))  min(Y) max(Y) 2;...                mean(cellN(:,org))  min(Y) max(Y) 0.1;...                median(cellN(:,org))  min(Y) max(Y) 0.1;...                mean(cellN(:,org))  min(Y) max(Y) 0.0;...                median(cellN(:,org))  min(Y) max(Y) 0.0];        for ip=1:size(par0,1)% optimization at different initial conditions.            [par(ip,:),fval(ip),exitflag(ip)] = fmincon(@sigmoidFittingError2,par0(ip,:),[],[],[],[],[],[],[],options,cellN(:,org),Y);            CC(ip)=corr(sigmoid(cellN(:,org),par(ip,:)),Y);            color=(randi(256,1,3)-1)/255;            plot(X,sigmoid(X,par0(ip,:)),'-.','Color',color,'LineWidth',0.5)            plot(X,sigmoid(X,par(ip,:)),'-','Color',color,'LineWidth',0.5)        end        minI=find(fval==min(fval));        if length(minI)>1            minI=minI(1);            %find(CC(minI)==min(CC(minI)))            %min(CC(minI))        end        parf=par(minI,:);        parOut(org,:)=parf;        if (org==size(cellN,2))            save(matfilename,"parOut");        end    else        load(matfilename)        parf=parOut(org,:);    end        % procedure to evaluate the range of residual cells in which the slope    % is moderate    dsig=derivSigmoid(X,parf);    thre=10;    funcCell=X((atand(dsig)>0+thre) & (atand(dsig)<90-thre));    funcCellRange=range(funcCell);    fill(funcCell([1 end end 1]),[min(Y) min(Y) max(Y) max(Y)],'r','EdgeColor','none','FaceAlpha',0.4)    plot(X,sigmoid(X,parf),'r','LineWidth',2);    %plot(funcCell,sigmoid(funcCell,parf),'r','LineWidth',4)        %plot(funcCell,atand(derivSigmoid(X,parf)),'r--')    axis equal            % For each data point I compute the closes point on the sigmoid.    for p=1:length(Y)        % function computing the 2D distance between the point        % (cellN(p,org),Y(p)) and the sigmoid.        fun=@(x)((cellN(p,org)-x)^2+(sigmoid(x,parf)-Y(p))^2);        [~,s0]=min((X-cellN(p,org)).^2+(sigmoid(X,parf)-Y(p)).^2);        [oX(p),mindist(p),exitflgX(p)]=fminsearch(fun,X(s0));        if fun(X(s0))<fun(oX(p))            keyboard        end        SigmPoint(p,:)=[ oX(p), sigmoid(oX(p),parf)];        plot([SigmPoint(p,1) cellN(p,org)],[SigmPoint(p,2) Y(p)],'k-','LineWidth',0.01)    end    MSD=mean((SigmPoint(:,1)-cellN(:,org)).^2+(SigmPoint(:,2)-Y(:)).^2);    %MSE=min(fval)/length(Y);    %compute the vaf = (1 - var(y-yhat)/var(y))*100    vaf = (1 - var(Y-sigmoid(cellN(:,org),parf))/var(Y))*100; % I'm not convinced by this equation because it does not take into account the noise on the cell#    %vaf2 = (1 - MSE/var(Y))*100;    %vaf3 = (1 - var((SigmPoint(:,1)-cellN(:,org)).^2+(SigmPoint(:,2)-Y(:)).^2)/var((cellN(:,org)-0).^2+(Y(:)-0).^2) )*100    %vaf4 = (1 - var((SigmPoint(:,1)-cellN(:,org)).^2+(SigmPoint(:,2)-Y(:)).^2)/var((cellN(:,org)-mean(cellN(:,org))).^2+(Y(:)-mean(Y)).^2) )*100    R2 = 1 - sum((Y-sigmoid(cellN(:,org),parf)).^2)/sum((Y-mean(Y)).^2);        temp1= SigmPoint-repmat(mean(SigmPoint),[size(SigmPoint,1) 1]);    varL=var(temp1(:,1)+temp1(:,2));    Point=[cellN(:,org) Y];    temp2= Point-repmat(mean(Point),[size(Point,1) 1]);    varP=var(temp2(:,1)+temp2(:,2));    vaf5=varL/varP*100;    %R2b=1-sum((sum((SigmPoint-Point).^2,2)).^2)/sum((sum((Point-mean(Point)).^2,2)).^2);    %plot(SigmPoint(:,1),SigmPoint(:,2),'gx')-Point    %plot(Point(:,1),Point(:,2),'bx')    mdev=mean(sqrt(sum((SigmPoint-Point).^2,2)));    Sdev2=sum(sum((SigmPoint-Point).^2,2));    dist=[];    distL=[];    mPoint=mean(Point);    mSigmPoint=mean(SigmPoint);        R2b=sum(sum((SigmPoint-mSigmPoint).^2,2))/sum(sum((Point-mPoint).^2,2))*100;    for p=1:length(Point)-1        for q=(p+1):length(Point)            dist=[dist sqrt(sum((Point(p,:)-Point(q,:)).^2,2))];            distL=[distL sqrt(sum((SigmPoint(p,:)-SigmPoint(q,:)).^2,2))];        end    end        vaf6=(1-mdev/mean(dist))*100;    vaf6b=(1-Sdev2/sum((sum((Point-mean(Point)).^2,2)).^2))*100;    vaf7=mean(distL)/mean(dist)*100;    title([Organ_name{org} ' RMSD=' num2str(sqrt(MSD),2) ' R2=' num2str(R2,2) ' R2_{2D}=' num2str(R2b,2) ' FunctCellRange=' num2str(funcCellRange,2) ])%'% VAF7=' num2str(vaf7,2) '% X0=' num2str(parf(1),2) ' k=' num2str(parf(4),3)    if org==1; ylabel(bahav_name);end    xlabel(['residual ' cell_Name])endsavefig(['sigmoidInterp - ' cell_Name ' vs ' bahav_name '.fig'])end%%%%%%%%%%%%%%%%%%%%%%%%%%%%%%%%%%%%%%%%%%%%%%%%%%%%%%%%%%%%%%%%%%%%%%%%%%%%%%%%%%%%%%%%%%%%%%%%%%%%%%%%%%%%%%%%%%function [parOut]=optimalSigmoidFitting22D(cellN1,cellN2,Y,Organ_name,cell1_Name,cell2_Name,bahav_name,matfilename)options = optimoptions('fmincon');options = optimset('Display','iter');%,'Algorithm','sqp'%options.MaxFunctionEvaluations =4*10^4;set(0,'units','pixels')  Pix_SS = get(0,'screensize');figure('Name',['sigmoidInterp - ' cell1_Name ' & ' cell2_Name ' vs ' bahav_name],'Units', 'pixels','Position',[0 0 Pix_SS(3)-20 floor((Pix_SS(4)-105)/2)]);%figure('Name',['sigmoidInterp - ' cell_Name ' vs ' bahav_name],'Position',[0 floor((Pix_SS(4)-105)/2)+1 Pix_SS(3) floor((Pix_SS(4)-105)/2)]);for org=1:size(cellN1,2) %organs    subplot(2,3,org);hold on    plot(cellN1(:,org),Y,'o')    subplot(2,3,org+3);hold on    plot(cellN2(:,org),Y,'o')    X1=min(cellN1(:,org)):max(cellN1(:,org));    X2=min(cellN2(:,org)):max(cellN2(:,org));            %X01                    ymin1       ymax1       K1                                                          %X02                    ymin2       ymax2       K2    par0 = [mean(cellN1(:,org))     min(Y)      max(Y)      (max(Y)-min(Y))/(max(cellN1(:,org))-min(cellN1(:,org)))     mean(cellN2(:,org))      min(Y)      max(Y)      (max(Y)-min(Y))/(max(cellN2(:,org))-min(cellN2(:,org)));...            median(cellN1(:,org))   min(Y)      max(Y)      (max(Y)-min(Y))/(max(cellN1(:,org))-min(cellN1(:,org)))     median(cellN2(:,org))   min(Y)      max(Y)      (max(Y)-min(Y))/(max(cellN2(:,org))-min(cellN2(:,org)));...            0                       min(Y)      max(Y)      (max(Y)-min(Y))/(max(cellN1(:,org))-min(cellN1(:,org)))     0                       min(Y)      max(Y)      (max(Y)-min(Y))/(max(cellN2(:,org))-min(cellN2(:,org)));...            100                     min(Y)      max(Y)      (max(Y)-min(Y))/(max(cellN1(:,org))-min(cellN1(:,org)))     100                     min(Y)      max(Y)      (max(Y)-min(Y))/(max(cellN2(:,org))-min(cellN2(:,org)));...%             mean(cellN(:,org))  min(Y) max(Y) 2;...            median(cellN1(:,org))   min(Y)      max(Y)      2                                                           median(cellN2(:,org))   min(Y)      max(Y)      2;...%             mean(cellN(:,org))  min(Y) max(Y) 0.1;...            median(cellN1(:,org))   min(Y)      max(Y)      0.1                                                         median(cellN2(:,org))   min(Y)      max(Y)      0.1;...%             mean(cellN(:,org))  min(Y) max(Y) 0.0;...            median(cellN1(:,org))   min(Y)      max(Y)      0.0                                                         median(cellN2(:,org))   min(Y)      max(Y)      0.0;...            ];        if ~exist(matfilename, "file")        for ip=1:size(par0,1)% optimization at different initial conditions.            [par(ip,:),fval(ip),exitflag(ip)] = fmincon(@sigmoidFittingError22D,par0(ip,:),[],[],[],[],[],[],[],options,cellN1(:,org),cellN2(:,org),Y);            CC1(ip)=corr(sigmoid(cellN1(:,org),par(ip,1:4)),Y);            CC2(ip)=corr(sigmoid(cellN2(:,org),par(ip,(1:4)+4)),Y);            color=(randi(256,1,3)-1)/255;            subplot(2,3,org);            plot(X1,sigmoid(X1,par0(ip,1:4)),'-.','Color',color,'LineWidth',0.5)            plot(X1,sigmoid(X1,par(ip,1:4)),'-','Color',color,'LineWidth',0.5)            subplot(2,3,org+3);            plot(X2,sigmoid(X2,par0(ip,5:8)),'-.','Color',color,'LineWidth',0.5)            plot(X2,sigmoid(X2,par(ip,5:8)),'-','Color',color,'LineWidth',0.5)        end        minI=find(fval==min(fval));        if length(minI)>1            minI=minI(1);            %find(CC(minI)==min(CC(minI)))            %min(CC(minI))        end        parf=par(minI,:);        parOut(org,:)=parf;        if (org==3)            save(matfilename,'parOut');        end    else        load(matfilename)        parf=parOut(org,:);    end    for p=1:length(Y)        % function computing the 2D distance between the point        % (cellN(p,org),Y(p)) and the sigmoid.        fun1=@(x)((cellN1(p,org)-x)^2+(sigmoid(x,parf(1:4))-Y(p))^2);        [~,s0]=min((X1-cellN1(p,org)).^2+(sigmoid(X1,parf(1:4))-Y(p)).^2);        [oX1(p),mindist1(p),exitflgX1(p)]=fminsearch(fun1,X1(s0));        SigmPoint1(p,:)=[ oX1(p), sigmoid(oX1(p),parf(1:4))];        subplot(2,3,org);        plot([SigmPoint1(p,1) cellN1(p,org)],[SigmPoint1(p,2) Y(p)],'k-','LineWidth',0.01)        fun2=@(x)((cellN2(p,org)-x)^2+(sigmoid(x,parf(5:8))-Y(p))^2);        [~,s0]=min((X2-cellN2(p,org)).^2+(sigmoid(X2,parf(5:8))-Y(p)).^2);        [oX2(p),mindist2(p),exitflgX2(p)]=fminsearch(fun2,X2(s0));        SigmPoint2(p,:)=[ oX2(p), sigmoid(oX2(p),parf(5:8))];        subplot(2,3,org+3);        plot([SigmPoint2(p,1) cellN2(p,org)],[SigmPoint2(p,2) Y(p)],'k-','LineWidth',0.01)    end    MSD1=mean((SigmPoint1(:,1)-cellN1(:,org)).^2+(SigmPoint1(:,2)-Y(:)).^2);    MSD2=mean((SigmPoint2(:,1)-cellN2(:,org)).^2+(SigmPoint2(:,2)-Y(:)).^2);    subplot(2,3,org);    plot(X1,sigmoid(X1,parf(1:4)),'r','LineWidth',2)    title([Organ_name{org} ' RMSD=' num2str(sqrt(MSD1),2)  ' X0=' num2str(parf(1),2) ' k=' num2str(parf(4),3)])    if org==1; ylabel(bahav_name);end    xlabel(['residual ' cell1_Name])    axis equal    subplot(2,3,org+3);    plot(X2,sigmoid(X2,parf(5:8)),'r','LineWidth',2)    title([Organ_name{org} ' RMSD=' num2str(sqrt(MSD2),2)  ' X0=' num2str(parf(5),2) ' k=' num2str(parf(8),3)])    if org==1; ylabel(bahav_name);end    xlabel(['residual ' cell2_Name])    axis equalendsavefig(['sigmoidInterp - ' cell1_Name ' & ' cell2_Name ' vs ' bahav_name])end%%%%%%%%%%%%%%%%%%%%%%%%%%%%%%%%%%%%%%%%%%%%%%%%%%%%%%%%%%%%%%%%%%%%%%%%%%%%%%%%%%%%%%%%%%%%%%%%%%%%%%%%%%%%%%%%%%function err=sigmoidFittingError2(param,X,Y)Xrange=floor(min(X)):ceil(max(X));sig=sigmoid(Xrange,param);dist=zeros(length(X),length(Xrange));for p=1:length(X)    for ncell=Xrange        dist(p,Xrange==ncell)=(X(p)-ncell)^2+(Y(p)-sig(Xrange==ncell))^2;    endenderr=sum(min(dist,[],2));    %err=(mean((sigmoid(X,param)-Y).^2));endfunction err=sigmoidFittingError(param,X,Y)    err=(mean((sigmoid(X,param)-Y).^2));endfunction y=sigmoid(x,param)x0=param(1);ymin=param(2);ymax=param(3);k=param(4);yt= (exp(k*(x-x0))-exp(-k*(x-x0)))./(exp(k*(x-x0))+exp(-k*(x-x0)));y=(yt+1)*(ymax-ymin)/2+ymin;endfunction dy=derivSigmoid(x,param)x0=param(1);ymin=param(2);ymax=param(3);k=param(4);yt= (exp(k*(x-x0))-exp(-k*(x-x0)))./(exp(k*(x-x0))+exp(-k*(x-x0)));dy=(ymax-ymin)/2*k*(1-(yt).^2);end%%%%%%%%%%%%%%%%%%%%%%%%%%%%%%%%%%%%%%%%%%%%%%%%%%%%%%%%%%%%%%%%%%%%%%%%%%%%%%%%%%%%%%%%%%%%%%%%%%%%%%%%%%%%%%%%%%function err=sigmoidFittingError22D(param,X1,X2,Y)X1range=floor(min(X1)):ceil(max(X1));X2range=floor(min(X2)):ceil(max(X2));sig1=sigmoid(X1range,param(1:4));sig2=sigmoid(X2range,param(5:8));dist1=zeros(length(X1),length(X1range));dist2=zeros(length(X2),length(X2range));for p=1:length(X1)    for ncell=X1range        dist1(p,X1range==ncell)=(X1(p)-ncell)^2+(Y(p)-sig1(X1range==ncell))^2;    endendfor p=1:length(X2)    for ncell=X2range        dist2(p,X2range==ncell)=(X2(p)-ncell)^2+(Y(p)-sig2(X2range==ncell))^2;    endenderr1=sum(min(dist1,[],2));err2=sum(min(dist2,[],2));err3=(sig1(1)-sig2(1))^2+(sig1(end)-sig2(end))^2;err=err1+err2+100*err3;    %err=(mean((sigmoid(X,param)-Y).^2));end%%%%%%%%%%%%%%%%%%%%%%%%%%%%%%%%%%%%%%%%%%%%%%%%%%%%%%%%%%%%%%%%%%%%%%%%%%%%%%%%%%%%%%%%%%%%%%%%%%%%%%%%%%%%%%%%%%% function err=sigmoidFittingError(param,X,Y)%     err=(mean((sigmoid(X,param)-Y).^2));% end% % function y=sigmoid(x,param)% x0=param(1);% ymin=param(2);% ymax=param(3);% k=param(4);% % yt= (exp(k*(x-x0))-exp(-k*(x-x0)))./(exp(k*(x-x0))+exp(-k*(x-x0)));% y=(yt+1)*(ymax-ymin)/2+ymin;% endfunction optimalSigmoidFitting3D(cellN1,cellN2,Y,Organ_name,cell1_Name,cell2_Name,bahav_name)options = optimoptions('fmincon');options = optimset('Display','iter');%,'Algorithm','sqp'%options.MaxFunctionEvaluations =4*10^4;set(0,'units','pixels')  Pix_SS = get(0,'screensize');figure('Name',['sigmoidInterp - ' cell1_Name ' & ' cell2_Name ' vs ' bahav_name],'Units', 'pixels','Position',[0 0 Pix_SS(3)-20 floor((Pix_SS(4)-105)/2)]);%figure('Name',['sigmoidInterp - ' cell_Name ' vs ' bahav_name],'Position',[0 floor((Pix_SS(4)-105)/2)+1 Pix_SS(3) floor((Pix_SS(4)-105)/2)]);for org=1:size(cellN1,2) %organs    subplot(1,3,org);hold on    plot3(cellN1(:,org),cellN2(:,org),Y,'o')    X1 = linspace(min(cellN1(:,org)), max(cellN1(:,org)),100);    X2 = linspace(min(cellN2(:,org)), max(cellN2(:,org)),100);            %X01                    X02                        K1                                                           K2                                                              ymin    ymax    par0 = [mean(cellN1(:,org))     mean(cellN2(:,org))        (max(Y)-min(Y))/(max(cellN1(:,org))-min(cellN1(:,org)))    (max(Y)-min(Y))/(max(cellN2(:,org))-min(cellN2(:,org)))       min(Y) max(Y)   ;...            median(cellN1(:,org))   median(cellN2(:,org))      (max(Y)-min(Y))/(max(cellN1(:,org))-min(cellN1(:,org)))      (max(Y)-min(Y))/(max(cellN2(:,org))-min(cellN2(:,org)))     min(Y) max(Y) ;...%             0                       0min(Y) max(Y) (max(Y)-min(Y))/(max(cellN(:,org))-min(cellN(:,org)));...%             100  min(Y) max(Y) (max(Y)-min(Y))/(max(cellN(:,org))-min(cellN(:,org)));...%             mean(cellN(:,org))  min(Y) max(Y) 2;...%             median(cellN(:,org))  min(Y) max(Y) 2;...%             mean(cellN(:,org))  min(Y) max(Y) 0.1;...%             median(cellN(:,org))  min(Y) max(Y) 0.1;...%             mean(cellN(:,org))  min(Y) max(Y) 0.0;...%             median(cellN(:,org))  min(Y) max(Y) 0.0...            ];        for ip=1:size(par0,1)% optimization at different initial conditions.        [par(ip,:),fval(ip),exitflag(ip)] = fmincon(@sigmoidFittingError3D,par0(ip,:),[],[],[],[],[],[],[],options,cellN1(:,org),cellN2(:,org),Y);        CC(ip)=corr(sigmoid3D(cellN1(:,org),cellN2(:,org),par(ip,:)),Y);        color=(randi(256,1,3)-1)/255;        plot3(X1,X2,sigmoid3D(X1,X2,par0(ip,:)),'-.','Color',color,'LineWidth',0.5)        plot3(X1,X2,sigmoid3D(X1,X2,par(ip,:)),'-','Color',color,'LineWidth',0.5)    end    minI=find(fval==min(fval));    if length(minI)>1        minI=minI(1);        %find(CC(minI)==min(CC(minI)))        %min(CC(minI))    end    parf=par(minI,:);    plot3(X1,X2,sigmoid3D(X1,X2,parf),'r','LineWidth',2)    title([Organ_name{org} ' RMSE=' num2str(sqrt(min(fval)),2) ' R=' num2str(CC(fval==min(fval)),2) ' X0=' num2str(parf(1),2) ' k=' num2str(parf(4),3)])    if org==1; zlabel(bahav_name);end    xlabel(['residual ' cell1_Name])    ylabel(['residual ' cell2_Name])endendfunction err=sigmoidFittingError3D(param,X1,X2,Y)% compute the fitting error of a sigmoid line in 3D spaceXrange1=linspace(floor(min(X1)),ceil(max(X1)),100);%floor(min(X1)):ceil(max(X1));Xrange2=linspace(floor(min(X2)),ceil(max(X2)),100);%floor(min(X2)):ceil(max(X2));sig=sigmoid3D(Xrange1,Xrange2,param);dist=zeros(length(X1),length(Xrange1));for p=1:length(X1)    for ncell=Xrange1        dist(p,Xrange1==ncell)=(X1(p)-ncell)^2+(X2(p)-ncell)^2+(Y(p)-sig(Xrange1==ncell))^2;    endenderr=sum(min(dist,[],2));    %err=(mean((sigmoid(X,param)-Y).^2));endfunction y=sigmoid3D(x1,x2,param)%Compute a sigmoid line in 3D spacex01=param(1);x02=param(2);k1=param(3);k2=param(4);ymin=param(5);ymax=param(6);yt1= (exp(k1*(x1-x01))-exp(-k1*(x1-x01)))./(exp(k1*(x1-x01))+exp(-k1*(x1-x01)));yt2= (exp(k2*(x2-x02))-exp(-k2*(x2-x02)))./(exp(k2*(x2-x02))+exp(-k2*(x2-x02)));y=((yt1+yt2)/2+1)*(ymax-ymin)/2+ymin;endfunction plot3DFunctionCells(Behav,nHC1Comb, nHC2Comb, Dose_IDPN, dose_list,parHC1_Behav,parHC2_Behav)Organ_name={'CRISTA','UTRICLE','SACCULE'};DoseColor=[0 0.4470 0.7410; 0.8500 0.3250 0.0980; 0.9290 0.6940 0.1250; 0.4940 0.1840 0.5560; 0.4660 0.6740 0.1880; 0.3010 0.7450 0.9330; 0.6350 0.0780 0.1840];figure("Name",'Correlation nTailLiftEND-HC1-HC2 per organ');for org=1:3    %[rhoP{org}, pvalP{org}]=corr(nHC1Comb(:,org), nHC2Comb(:,org));    %[rhoS{org}, pvalS{org}]=corr(nHC1Comb(:,org), nHC2Comb(:,org),'Type','Spearman');    subplot(1,3,org);hold on    for d=1:length(dose_list)        h(d)=plot3(nHC1Comb(Dose_IDPN==dose_list(d),org),nHC2Comb(Dose_IDPN==dose_list(d),org),Behav(Dose_IDPN==dose_list(d)),'o','Color',DoseColor(d,:),'MarkerFaceColor',DoseColor(d,:));        plot3(nHC1Comb(Dose_IDPN==dose_list(d),org),nHC2Comb(Dose_IDPN==dose_list(d),org),-120+0*Behav(Dose_IDPN==dose_list(d)),'x','Color',DoseColor(d,:));        plot3(150+0*nHC1Comb(Dose_IDPN==dose_list(d),org),nHC2Comb(Dose_IDPN==dose_list(d),org),Behav(Dose_IDPN==dose_list(d)),'x','Color',DoseColor(d,:));        plot3(nHC1Comb(Dose_IDPN==dose_list(d),org),150+0*nHC2Comb(Dose_IDPN==dose_list(d),org),Behav(Dose_IDPN==dose_list(d)),'x','Color',DoseColor(d,:));        pointList=find(Dose_IDPN==dose_list(d));%         for p=1:length(pointList)%             plot3(nHC1Comb(pointList(p),org)*[1 1],nHC2Comb(pointList(p),org)*[1 1],[Behav(pointList(p)) -120],'Color',DoseColor(d,:),'LineWidth',0.01);%             plot3([150 nHC1Comb(pointList(p),org)],nHC2Comb(pointList(p),org)*[1 1],Behav(pointList(p))*[1 1],'Color',DoseColor(d,:),'LineWidth',0.01);%             plot3(nHC1Comb(pointList(p),org)*[1 1],[150 nHC2Comb(pointList(p),org)],Behav(pointList(p))*[1 1],'Color',DoseColor(d,:),'LineWidth',0.01);%         end%             end    %axis equal    xlim([-5 150])    ylim([-5 150])    xlabel('% residual HC1')    ylabel('% residual HC2')    zlabel('Behaviour')    grid on    title([Organ_name{org}])%,  ' rhoP=', num2str(rhoP{org},2), ', p=', num2str(pvalP{org},2),  ' rhoS=', num2str(rhoS{org},2), ', p=', num2str(pvalS{org},2)])    view([-100 -100 50])    % plot the 3D sigmoid line by "intersection of the two 2D lines    if exist("parHC1_Behav","var") && exist("parHC2_Behav","var")        X1=linspace(floor(min(nHC1Comb(:,org))),ceil(max(nHC1Comb(:,org))),100);        Y1=ones(size(X1))*150;        Z1=sigmoid(X1,parHC1_Behav(org,:));        plot3(X1,Y1,Z1,'r','LineWidth',2)            Y2=linspace(floor(min(nHC2Comb(:,org))),ceil(max(nHC2Comb(:,org))),100);        X2=ones(size(Y2))*150;        Z2=sigmoid(Y2,parHC2_Behav(org,:));        plot3(X2,Y2,Z2,'r','LineWidth',2)        Z3=linspace(min(Z1(1),Z2(1)),max(Z1(end),Z2(end)),500);        for c=1:length(Z3)            fun1=@(x)(sigmoid(x,parHC1_Behav(org,:))-Z3(c))^2;            [~,s0]=min((Z1-Z3(c)).^2);            [X3(c),fvalX(c),exitflgX(c)]=fminsearch(fun1,X1(s0));            fun2=@(x)(sigmoid(x,parHC2_Behav(org,:))-Z3(c))^2;                        [~,s0]=min((Z2-Z3(c)).^2);            [Y3(c),fvalY(c),exitflgY(c)]=fminsearch(fun2,Y2(s0));                    end        plot3(X3,Y3,Z3,'-xb','LineWidth',2)    endendlegend(h,string(dose_list))end%%%%%%%%%%%%%%%%%%%%%%%%%%%%%%%%%%%%%%%%%%%%%%%%%%%%%%%%%%%%%%%%%%%%%%%%%%%function plot3DFunctionCells2(Behav,nHC1Comb, nHC2Comb, Dose_IDPN, dose_list,parHC1HC2_Behav,parHC1HC2,parHC2HC1)Organ_name={'CRISTA','UTRICLE','SACCULE'};DoseColor=[0 0.4470 0.7410; 0.8500 0.3250 0.0980; 0.9290 0.6940 0.1250; 0.4940 0.1840 0.5560; 0.4660 0.6740 0.1880; 0.3010 0.7450 0.9330; 0.6350 0.0780 0.1840];figure("Name",'Correlation nTailLiftEND-HC1-HC2 per organ');for org=1:3    %[rhoP{org}, pvalP{org}]=corr(nHC1Comb(:,org), nHC2Comb(:,org));    %[rhoS{org}, pvalS{org}]=corr(nHC1Comb(:,org), nHC2Comb(:,org),'Type','Spearman');    subplot(1,3,org);hold on    for d=1:length(dose_list)        h(d)=plot3(nHC1Comb(Dose_IDPN==dose_list(d),org),nHC2Comb(Dose_IDPN==dose_list(d),org),Behav(Dose_IDPN==dose_list(d)),'o','Color',DoseColor(d,:),'MarkerFaceColor',DoseColor(d,:));        plot3(nHC1Comb(Dose_IDPN==dose_list(d),org),nHC2Comb(Dose_IDPN==dose_list(d),org),-120+0*Behav(Dose_IDPN==dose_list(d)),'x','Color',DoseColor(d,:));        plot3(150+0*nHC1Comb(Dose_IDPN==dose_list(d),org),nHC2Comb(Dose_IDPN==dose_list(d),org),Behav(Dose_IDPN==dose_list(d)),'x','Color',DoseColor(d,:));        plot3(nHC1Comb(Dose_IDPN==dose_list(d),org),150+0*nHC2Comb(Dose_IDPN==dose_list(d),org),Behav(Dose_IDPN==dose_list(d)),'x','Color',DoseColor(d,:));        pointList=find(Dose_IDPN==dose_list(d));%         for p=1:length(pointList)%             plot3(nHC1Comb(pointList(p),org)*[1 1],nHC2Comb(pointList(p),org)*[1 1],[Behav(pointList(p)) -120],'Color',DoseColor(d,:),'LineWidth',0.01);%             plot3([150 nHC1Comb(pointList(p),org)],nHC2Comb(pointList(p),org)*[1 1],Behav(pointList(p))*[1 1],'Color',DoseColor(d,:),'LineWidth',0.01);%             plot3(nHC1Comb(pointList(p),org)*[1 1],[150 nHC2Comb(pointList(p),org)],Behav(pointList(p))*[1 1],'Color',DoseColor(d,:),'LineWidth',0.01);%         end%             end    %axis equal    xlim([-5 150])    ylim([-5 150])    xlabel('% residual HC1')    ylabel('% residual HC2')    zlabel('Behaviour')    grid on    title([Organ_name{org}])%,  ' rhoP=', num2str(rhoP{org},2), ', p=', num2str(pvalP{org},2),  ' rhoS=', num2str(rhoS{org},2), ', p=', num2str(pvalS{org},2)])    view([-100 -100 50])    % plot the 3D sigmoid line by "intersection of the two 2D lines    if exist("parHC1HC2_Behav","var")         %        X1=linspace(floor(min(nHC1Comb(:,org))),ceil(max(nHC1Comb(:,org))),100);        Y1=ones(size(X1))*150;        Z1=sigmoid(X1,parHC1HC2_Behav(org,1:4));        plot3(X1,Y1,Z1,'r','LineWidth',2)            Y2=linspace(floor(min(nHC2Comb(:,org))),ceil(max(nHC2Comb(:,org))),100);        X2=ones(size(Y2))*150;        Z2=sigmoid(Y2,parHC1HC2_Behav(org,(1:4)+4));        plot3(X2,Y2,Z2,'r','LineWidth',2)        Z3=linspace(min(Z1(1),Z2(1)),max(Z1(end),Z2(end)),500);        for c=1:length(Z3)            fun1=@(x)((sigmoid(x,parHC1HC2_Behav(org,1:4))-Z3(c))^2);% + (sigmoid(sigmoid(x,parHC1HC2(org,:)),parHC1HC2_Behav(org,(1:4)+4))-Z3(c))^2);            [~,s0]=min((Z1-Z3(c)).^2);            [X3(c),fvalX(c),exitflgX(c)]=fminsearch(fun1,X1(s0));            fun2=@(x)((sigmoid(x,parHC1HC2_Behav(org,(1:4)+4))-Z3(c))^2);%+(sigmoid(sigmoid(x,parHC2HC1(org,:)),parHC1HC2_Behav(org,(1:4)))-Z3(c))^2);                        [~,s0]=min((Z2-Z3(c)).^2);            [Y3(c),fvalY(c),exitflgY(c)]=fminsearch(fun2,Y2(s0));                    end        plot3(X3,Y3,Z3,'-xb','LineWidth',2)%         X4=linspace(floor(min(nHC1Comb(:,org))),ceil(max(nHC1Comb(:,org))),100);%         Y4=sigmoid(X4,parHC1HC2(org,:));%         Z4a=sigmoid(X4,parHC1HC2_Behav(org,1:4));%         Z4b=sigmoid(Y4,parHC1HC2_Behav(org,5:8));%         Z4=(Z4a+Z4b)/2;%         plot3(X4,Y4,Z4,'-xc','LineWidth',2)%         %plot3(X4,Y4,Z4b,'-xm','LineWidth',2)%         %plot3(X4,Y4,Z4a,'-xg','LineWidth',2)%         Y5=linspace(floor(min(nHC2Comb(:,org))),ceil(max(nHC2Comb(:,org))),100);%         X5=sigmoid(Y5,parHC2HC1(org,:));%         Z5a=sigmoid(X5,parHC1HC2_Behav(org,1:4));%         Z5b=sigmoid(Y5,parHC1HC2_Behav(org,5:8));%         Z5=(Z5a+Z5b)/2;%         plot3(X5,Y5,Z5,'-xm','LineWidth',2)   endendlegend(h,string(dose_list))endfunction [parOut]=SigmoidFittinHC1HC2 (cellN1,cellN2,Organ_name,cell1_Name,cell2_Name,matfilename)options = optimoptions('fmincon');options = optimset('Display','iter');%,'Algorithm','sqp'set(0,'units','pixels')  Pix_SS = get(0,'screensize');figure('Name',['sigmoidInterp - ' cell1_Name ' vs ' cell2_Name ],'Units', 'pixels','Position',[0 0 Pix_SS(3)-20 floor((Pix_SS(4)-105)/2)]);for org=1:size(cellN1,2) %organs    subplot(1,3,org);hold on    plot(cellN1(:,org),cellN2(:,org),'o')    X=min(cellN1(:,org)):max(cellN1(:,org));    %X2=min(cellN2(:,org)):max(cellN2(:,org));            %X01                    ymin1       ymax1       K1                                                              par0 = [mean(cellN1(:,org))     min(cellN2(:,org))      max(cellN2(:,org))      (max(cellN2(:,org))-min(cellN2(:,org)))/(max(cellN1(:,org))-min(cellN1(:,org)))     ;...            median(cellN1(:,org))   min(cellN2(:,org))      max(cellN2(:,org))      (max(cellN2(:,org))-min(cellN2(:,org)))/(max(cellN1(:,org))-min(cellN1(:,org)))     ;...            0                       min(cellN2(:,org))      max(cellN2(:,org))      (max(cellN2(:,org))-min(cellN2(:,org)))/(max(cellN1(:,org))-min(cellN1(:,org)))     ;...            100                     min(cellN2(:,org))      max(cellN2(:,org))      (max(cellN2(:,org))-min(cellN2(:,org)))/(max(cellN1(:,org))-min(cellN1(:,org)))     ;...%             mean(cellN(:,org))  min(Y) max(Y) 2;...            median(cellN1(:,org))   min(cellN2(:,org))      max(cellN2(:,org))      2                                                           ;...%             mean(cellN(:,org))  min(Y) max(Y) 0.1;...            median(cellN1(:,org))   min(cellN2(:,org))      max(cellN2(:,org))      0.1                                                         ;...%             mean(cellN(:,org))  min(Y) max(Y) 0.0;...            median(cellN1(:,org))   min(cellN2(:,org))      max(cellN2(:,org))      0.0                                                         ;...            ];    if ~exist(matfilename, "file")        for ip=1:size(par0,1)% optimization at different initial conditions.            [par(ip,:),fval(ip),exitflag(ip)] = fmincon(@sigmoidFittingError2,par0(ip,:),[],[],[],[],[],[],[],options,cellN1(:,org),cellN2(:,org));            %CC(ip)=corr(sigmoid(cellN(:,org),par(ip,:)),Y);            color=(randi(256,1,3)-1)/255;            plot(X,sigmoid(X,par0(ip,:)),'-.','Color',color,'LineWidth',0.5)            plot(X,sigmoid(X,par(ip,:)),'-','Color',color,'LineWidth',0.5)        end        minI=find(fval==min(fval));        if length(minI)>1            minI=minI(1);            %find(CC(minI)==min(CC(minI)))            %min(CC(minI))        end        parf=par(minI,:);        parOut(org,:)=parf;        if (org==3)            save(matfilename,"parOut");        end    else        load(matfilename)        parf=parOut(org,:);    end    plot(X,sigmoid(X,parf),'r','LineWidth',2)    axis equal    xlabel(cell1_Name)    ylabel(cell2_Name)    title(Organ_name{org})    % For each data point I compute the closes point on the sigmoid.    for p=1:length(cellN2(:,org))        % function computing the 2D distance between the point        % (cellN(p,org),Y(p)) and the sigmoid.        fun=@(x)((cellN1(p,org)-x)^2+(sigmoid(x,parf)-cellN2(p,org))^2);        [~,s0]=min((X-cellN1(p,org)).^2+(sigmoid(X,parf)-cellN2(p,org)).^2);        [oX(p),mindist(p),exitflgX(p)]=fminsearch(fun,X(s0));        if fun(X(s0))<fun(oX(p))            keyboard        end        SigmPoint(p,:)=[ oX(p), sigmoid(oX(p),parf)];        plot([SigmPoint(p,1) cellN1(p,org)],[SigmPoint(p,2) cellN2(p,org)],'k-','LineWidth',0.01)    endendend
